# Flexible and scalable genotyping-by-sequencing strategies for population studies

**DOI:** 10.1186/1471-2164-15-979

**Published:** 2014-11-18

**Authors:** Christopher Heffelfinger, Christopher A Fragoso, Maria A Moreno, John D Overton, John P Mottinger, Hongyu Zhao, Joe Tohme, Stephen L Dellaporta

**Affiliations:** Department of Molecular, Cellular, and Developmental Biology, Yale University, New Haven, CT 06511 USA; Department of Computational Biology and Bioinformatics, Yale University, New Haven, CT 06520-8034 USA; Yale Center for Genome Analysis, Yale University, New Haven, CT 06516 USA; Regeneron Genetics Center, Regeneron, Tarrytown, NY 10591 USA; Department of Cell and Molecular Biology, University of Rhode Island, Kingston, RI 02881 USA; Agrobiodiversity Research Area, Centro Internacional de Agricultura Tropical (CIAT), A.A. 6713 Cali, Colombia

**Keywords:** Genotyping, GBS, Reduced representation sequencing, Population genomics, Trait mapping, Plant breeding, Agricultural genomics

## Abstract

**Background:**

Many areas critical to agricultural production and research, such as the breeding and trait mapping in plants and livestock, require robust and scalable genotyping platforms. Genotyping-by-sequencing (GBS) is a one such method highly suited to non-human organisms. In the GBS protocol, genomic DNA is fractionated via restriction digest, then reduced representation is achieved through size selection. Since many restriction sites are conserved across a species, the sequenced portion of the genome is highly consistent within a population. This makes the GBS protocol highly suited for experiments that require surveying large numbers of markers within a population, such as those involving genetic mapping, breeding, and population genomics. We have modified the GBS technology in a number of ways. Custom, enzyme specific adaptors have been replaced with standard Illumina adaptors compatible with blunt-end restriction enzymes. Multiplexing is achieved through a dual barcoding system, and bead-based library preparation protocols allows for in-solution size selection and eliminates the need for columns and gels.

**Results:**

A panel of eight restriction enzymes was selected for testing on B73 maize and Nipponbare rice genomic DNA. Quality of the data was demonstrated by identifying that the vast majority of reads from each enzyme aligned to restriction sites predicted *in silico.* The link between enzyme parameters and experimental outcome was demonstrated by showing that the sequenced portion of the genome was adaptable by selecting enzymes based on motif length, complexity, and methylation sensitivity. The utility of the new GBS protocol was demonstrated by correctly mapping several in a maize F_2_ population resulting from a B73 × Country Gentleman test cross.

**Conclusions:**

This technology is readily adaptable to different genomes, highly amenable to multiplexing and compatible with over forty commercially available restriction enzymes. These advancements represent a major improvement in genotyping technology by providing a highly flexible and scalable GBS that is readily implemented for studies on genome-wide variation.

**Electronic supplementary material:**

The online version of this article (doi:10.1186/1471-2164-15-979) contains supplementary material, which is available to authorized users.

## Background

Genome resequencing has emerged as the principal means for identifying both the genotypes of single individuals and genetic variation within populations or species. Methods such as whole genome and whole exome sequencing can generate data on large numbers of common and rare variants and discover previously uncharacterized variants. Further, population genomics via sequencing shows reduced ascertainment bias relative to microarrays and other *a posteriori* methods [1–3]. Improvements in sequencing chemistry, methodologies, hardware, and software have increased sequencing read quantity and length, improved multiplexing scalability, and added further robustness to genotyping calls [4, 5]. Associated bioinformatics have seen similar advancement in the filtering of false positives, imputation of missing data, and utilization of datasets for genomics [6–11]. In the course of these advances, two major avenues for genome resequencing have emerged: whole genome sequencing (WGS) and a variety of methods collectively referred to as reduced representation sequencing (RRS).

WGS methodologies attempt to query the entire genome in an as unbiased a manner as technically possible by constructing and sequencing libraries of randomly sheared genomic DNA. Millions of short reads are aligned to a reference genome to identify variants. While per-base error rate in most NGS methodologies is low, technical limitations, insufficient sequencing depth, and sequence and structural inaccuracies in the reference genomes can result in numerous errors [9]. Deep sequence coverage of overlapping reads can significantly reduce errors in variant calling. Hence, each position in the genome is represented by many overlapping reads on both strands of DNA that result in highly robust genotype calls and reduced errors from PCR, sequencing artifacts, and alignment errors. The amount of sequencing required to achieve high coverage, especially in large eukaryotic genomes such as many plants, can be prohibitively expensive. This restricts the application of high-coverage WGS-based genotyping. Therefore, WGS methods that rely on 20× to 30× coverage are preferred when attempting to identify sample specific variation or very limited numbers of samples in a population are available.

Low-coverage (LC) WGS is typically kept around 5× and, in some cases, less than 1× mean coverage per base for a given sample. LC-WGS reduces the cost and improves the ability to multiplex samples in a single sequencing run. Its limitation is the accuracy of variant calling due to incomplete genome coverage and the inability to distinguish variants and inherent errors. For instance, polymorphisms may be lost in a sample due to low coverage or subsequent filtering during computational steps. Errors introduced by PCR and sequencing may be misidentified as variants when coverage is low. Nevertheless, when a reference genome and sufficient samples are available to infer haplotype structure, statistical methods such as imputation may result in variant calling that rivals that produced by HC-WGS both in terms of quantity and accuracy for a fraction of the cost [9, 12–14]. Yet, without some form of cross-sample validation of variation, LC-WGS is at a disadvantage to high coverage sequencing.

A second category of genome resequencing can be collectively called reduced representation sequencing (RRS) methodologies. Quite simply, RRS methodologies reduce a genome’s complexity by enriching, separating, or eliminating a portion of the genome prior to sequencing. Some methods attempt to increase the informative fraction of the sequenced genome, such as exome sequencing [15, 16], while others ensure a consistent portion of the genome is retargeted for sequencing among samples [14, 17–22]. Exome sequencing, the most common RRS methodology, is based on oligonucleotide capture technologies, where short DNA fragments bind complementary targets of interest. Captured fragments are then isolated from the rest of the genome and sequenced. Large oligo capture arrays allow high specificity even when interrogating large genomic regions, such as the human exome. While this technology can be applied to almost any set of targets, initial implementation can be very costly and requires the genome of interest be well characterized.

Alternative RRS technologies are restriction-site associated DNA (RAD) sequencing [19], spin-off methods called double-digest RAD or ddRAD [23], 2b-RAD [24], and a related method called Genotyping-By-Sequencing or GBS [18]. These methods rely on an initial digest of sample DNA by restriction enzyme to reduce genome representation. The 2b-RAD method uses a Type IIb restriction enzyme, which cuts at two points to produce a fixed-size dsDNA fragment. In ddRAD, a second digest of gDNA by a different enzyme follows the first. In both RAD and ddRAD, a biotinylated adaptor specific to the initial enzyme captures DNA fragments of interest [19]. 2b-RAD uses size selection to capture fragments of interest. RAD technologies and GBS can be adapted to poorly characterized genomes, but lack the specificity to regions of interest of exome sequencing. In addition, much of the sequence will originate from non-informative, repetitive regions.

GBS is similar to RAD sequencing whereby a restriction enzyme digest of gDNA produces a size spectrum of DNA fragments. As restriction enzyme sites are reasonably fixed (barring polymorphism) within a species’ genome, homologous regions will produce size spectrums that are consistent between members of a population. Reduced representation is achieved by sequencing a small range of fragment sizes, rather than by capture of biotinylated adaptor. GBS can target as little as 2.3% of a genome for sequencing [18]. More importantly, this small portion remains sufficiently consistent across samples to produce comparative results even in highly diverse species [25], especially when other resources, such as NAM lines or a high quality reference genome, are available to guide calls. In maize, which has undergone extensive GBS-based research, there is approximately tenfold more inter-accession diversity than exists across the spectrum of human populations [26, 27]. This methodology is easily implemented, low cost, adaptable to poorly characterized genomes, and suitable for large-scale multiplexing of both library preparation and sequencing [18]. Interest in the GBS protocol has resulted in expansions to the original protocol and improved computational data filtering and imputation [28–31].

In spite of its popularity, several issues limit the adoption of GBS methodology. One key issue is the requirement of customized barcoded adaptors specific to a single restriction overhang sequence. This greatly reduces flexibility and increases the cost of implementation. Based on the GBS methodology, we have developed a novel approach to genotyping via restriction enzyme-based reduced representation library. This approach, which is compatible with all blunt-end restriction enzymes, is high-throughput, scalable to large sample sizes, and has a significantly lower cost to implement than other methods. The key, novel element in this standardized protocol is the incorporation of universal adaptors that are compatible with any blunt-end restriction enzyme. Supporting this change is the use of a low-cycle PCR-based dual indexing system that allows exceptional multiplexing of individual samples, and a simple bead based library preparation protocol that allows in solution reaction cleanup and size selection in microtiter plates. Our results demonstrate how enzymes can be selected to meet the needs of a given experiment and how informative sequences can be enriched by selecting enzymes that minimize repetitive and ambiguous reads. High levels of multiplexing and consistent genome representation can be achieved by utilizing enzymes with complex recognition motifs, while enzymes with simple motifs may better serve experiments requiring extensive variant identification. Finally, we show that genome size, repetitiveness, methylation status, and quality of the associated reference are all factors that may ultimately affect enzyme selection.

## Results

### Modifications to existing GBS methods

To improve the flexibility and scalability of GBS several modifications were incorporated into the protocol. The key modification was that by choosing restriction enzymes that generated blunt ends fragments rather than ones with staggered ends, the custom enzyme-specific adaptors used in the original protocol [18] could be replaced with standard Illumina Y-adaptors. This change removes the need for a costly end-repair step in the library preparation and enables the protocol to be compatible with a variety of enzymes. Supporting the switch to blunt-end enzymes and universal Illumina Y-adaptors, barcodes that were previously incorporated into custom adaptors were replaced with a primer-based method that adds dual indices, one to each end of an adaptor ligated DNA fragment, during a low-cycle PCR step [32]. Finally, a Solid Phase Reversible Immobilization (SPRI) [33] based library preparation allows for the entire protocol, including size-selection, to be done in microtiter plates, without the need for gels or columns [34]. The results of these modifications were significant reduction in cost, compatibility with a variety of blunt-end restriction enzymes, and a streamlined protocol that was adaptable to high throughput population genomic applications. The ability to choose restriction enzymes has several advantages as discussed later.

To test the robustness of these changes to the GBS methods, eight blunt end restriction enzymes were surveyed on two different plant reference genomes *Zea mays* B73 [35] and *Oryza sativa japonica* Nipponbare [36]. These genomes differ significantly in size, repetitive content, and methylated fraction. These eight multiplexed samples from each library were pooled and sequenced. Enzymes, motifs, and summary sequencing information are summarized in Table 1.

**Table 1:** **Enzyme summary statistics**

	MlyI	AluI	RsaI	DraI	EcoRV	StuI	HaeIII	HincII
Recognition Motif	GAGTC(N) ^5^/	AG/CT	GT/AC	TTT/AAA	GAT/ATC	AGG/CCT	GG/CC	GTY/RAC
				Maize				
Reads (2 × 75bp)	11,092,770	68,513,249	13,758,608	2,039,750	1,495,384	785,205	60,419,585	1,011,458
Fraction reads with correct motif	0.389	0.995	0.969	0.957	0.882	0.904	0.996	0.851
				Rice				
Reads (2 × 75bp)	2,970,049	73,426,557	7,953,490	7,181,944	498,460	415,512	35,197,321	526,507
Fraction reads with correct motif	0.375	0.995	0.994	0.996	0.946	0.971	0.998	0.890

### Validation of restriction motif in reads

A detailed assessment of the quality of data produced was performed. The first parameter tested was the quality of the sequenced fragments by confirming the appropriate restriction motif at the end of reads. All restriction enzymes, other than MlyI, tested in maize and rice had >80% and in most cases >90% of reads with the proper cutsite (Table 1). MlyI is a special case, as its non-palindromic recognition site is offset from its cleavage site, which results in the restriction motif being absent from 50% of the reads. Only 38.9% and 37.5% of the reads in maize and rice were observed with the proper MlyI motif, however.

### Paired versus unpaired sequencing tags

The modified GBS method produces minimal chimeric reads due to the dA-tailing step. Thus, it is highly suited to paired-end sequencing and associated data analysis. Paired-end reads are generally held to be more likely to align correctly to a genome than single end reads, both due to the increased amount of sequence and the distance between sequences. To evaluate the effect of paired versus single end reads on alignment, the mapping quality of reads was assessed. Mapping quality (MQ) is a measure of confidence in a given read alignment, given the information available in the reference genome. MQ is a Phred scaled value; a MQ of 20 indicates a 1 in 100 chance of misalignment, and a MQ of 30 indicates a 1 in 1000 chance. Reads that map equally well at multiple locations or fail to map at all are given mapping qualities of 0. For many experiments, alignments below a certain mapping quality, usually values of 20, 30 or 40, are filtered out.

**Figure 1:**
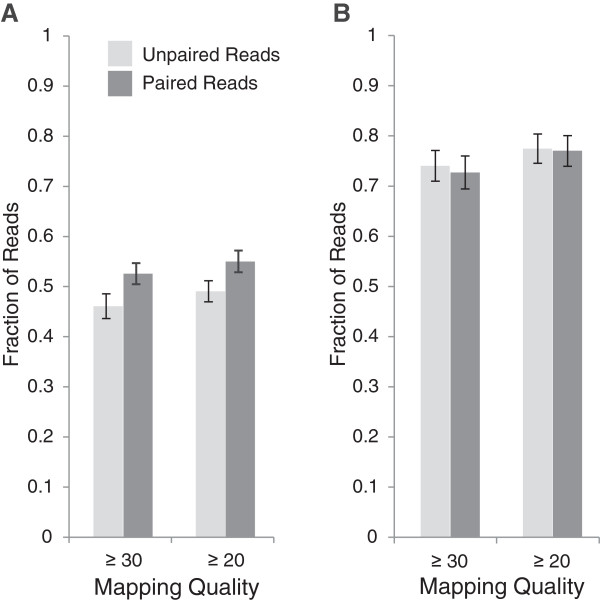
**Mapping quality differences between paired and unpaired reads.** The mean fraction and standard error of reads across all enzymes aligning at MQ ≥20 and MQ ≥30 was determined for **A)** maize and **B)** rice.

Sequences were retained as pairs or as “single tags” as in the original GBS protocol [29]. Paired reads are generally held to be more likely to map correctly than unpaired reads [37]. Sequences from each enzyme dataset were aligned as both paired and unpaired reads to the maize and rice reference genomes. The fraction of reads aligning at mapping quality MQ ≥20 and MQ ≥30 was then determined. In maize (Figure 1A), a significantly higher fraction of reads in the paired dataset aligned at MQ ≥20 (*p =*0.000, paired t-test) and MQ ≥30 (*p =*0.045, paired t-test). In rice (Figure 1B), there was no significant difference at MQ ≥20 (*p =*0.077, paired t-test) but a small significant decrease in the fraction of paired reads aligning at MQ ≥30 (*p =*0.045, paired t-test) compared to unpaired reads.

### Mapping quality

**Figure 2:**
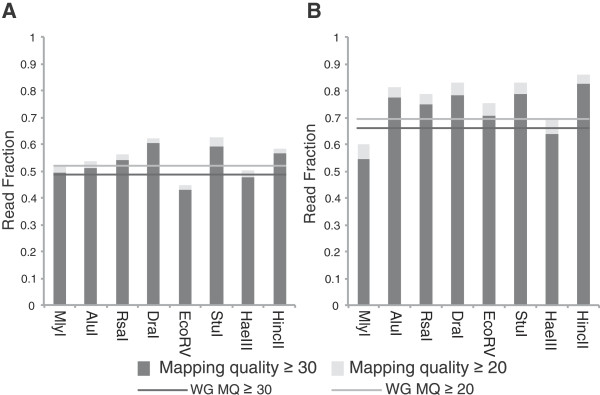
**Effect of enzyme choice on mapping quality.** Grey lines represent the fraction of reads with MQ ≥20 and MQ ≥30 of rice and maize whole genome samples consisting of one million paired-end reads truncated to 73 bp. The fraction of reads aligning at MQ ≥20 and MQ ≥30 in **A)** maize and **B)** rice were compared to WG samples.

A major source of data loss in GBS and many other next generation sequencing methodologies is the inability to align reads with sufficient confidence. To assess read alignment quality in the dataset, overall mapping quality of one million paired end reads was assessed at MQ ≥20 and MQ ≥30 for each enzyme in both maize and rice and compared to the MQ distribution of whole genome samples consisting of one million paired-end reads truncated to 73 bp. In maize (Figure 2A) HincII, StuI, and DraI all had MQ scores higher than the whole genome control (0.519 ≥ MQ 20, 0.480 ≥ MQ 30), while RsaI and EcoRV were lower. In rice (Figure 2B), the majority of enzymes had higher MQ scores than the whole genome dataset (0.697 ≥ MQ 20, 0.668 ≥ MQ 20), except for HaeIII, which was similar in value, and MlyI, which was considerably lower. These studies indicated that enzyme choice influences the proportion of reads that could be confidently aligned to the genome and utilized in downstream experiments.

### In silico site prediction

**Figure 3:**
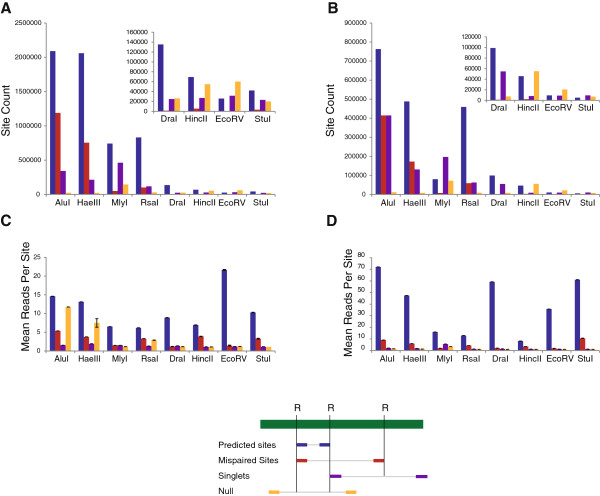
**Read alignments compared to digest sites predicted** ***in silico*** **.** An *in silico* digest of the *maize* and *rice* reference genomes identified predicted restriction sites for each enzyme. Following alignment to the genome, sequencing reads from each GBS dataset were categorized as “predicted” (blue) when the ends aligned to proximal restriction sites, “mispaired” (red) when the ends aligned to non-proximal restriction sites, “singlet” (purple) when only one end of a read aligned to a restriction site, and “null” (orange) when neither end of a read aligned to an *in silico* predicted restriction site. The number of each type of site with coverage (mapping quality (MQ) ≥20) was determined for both **A)** maize and **B)** rice. The mean coverage per site type (MQ ≥20) was also calculated in **C)** maize and **D)** rice for all enzymes.

A key goal of this project was to both be able to predict which sites would be covered by sequencing reads and to understand the factors affecting sequencing coverage. Simply quantifying each individual restriction site as having reads aligning to it or not would fail to distinguish between restriction sites that would reliably generate sequencing reads and restriction sites that generated spurious reads from singular events. An example of a singular event would be a restriction site that would not normally be covered due to the distance between it and proximal restriction sites occurring sufficiently close to the random end of a DNA strand to produce a suitable fragment for sequencing. Instead, sites were classified into four categories based on restriction sites identified *in silico* and the alignments of both ends of paired-end reads (Figure 3).

“Predicted” sites were defined as reads originating from proximal restriction sites. Reads aligning to non-proximal restriction sites were designated as “mispaired”. Paired reads with one end aligning to a restriction site and the other end aligning to no predicted restriction site were designated “singlets”. Reads that did not align to any predicted restriction site were identified as “null”. Only reads with a mapping quality (MQ) score ≥20, or a 99% chance of correct alignment, were included for further analysis.

We predicted that the vast majority of reads for all enzymes would originate from proximal restriction sites, which we designated as predicted sites. To test this, the alignments of actual reads (MQ ≥20) were compared to *in silico* digest predictions of the maize and rice reference genomes. In maize, between 80.9% and 94.8% of all actual reads with MQ ≥20 aligned to predicted sites, while in rice 71.3% to 94.8% of reads aligned to predicted sites (Table 2). In raw count of unique sites with sequencing coverage, predicted sites were the most common for all enzymes except EcoRV in maize (Figure 3A) and HincII in rice (Figure 3B). In terms of depth of coverage, predicted sites were the highest across all enzymes in both maize (Figure 3C) and rice (Figure 3D). The ultimate outcome of this analysis was the conclusion that proximal restriction sites are the origin of most sequenced reads. This provided us with a framework for the prediction of sequenced sites. This framework not only allowed us to predict what sites might be covered, but to compare the total set of predicted sites to the subset of sites with sequencing coverage to discover factors that influence site coverage.

**Table 2:** **Predicted site counts and coverage**

	MlyI	AluI	RsaI	DraI	EcoRV	StuI	HaeIII	HincII
				**Maize**				
Total predicted sites	3,326,697	8,886,974	4,870,173	894,567	427,268	515,556	7,667,926	1,376,427
Predicted sites 100-1000 bp	1,898,039	5,083,913	3,105,315	320,985	71,742	134,944	3,823,749	583,683
Predicted sites 100-400 bp	1,010,023	3,701,470	1,815,078	175,669	23,107	69,468	2,690,200	246,190
Predicted sites 100-200 bp	391,160	1,936,120	746,075	78,672	10,382	21,045	1,436,751	103,861
Total predicted sites covered*	0.2226	0.2350	0.1705	0.1514	0.0604	0.0818	0.2684	0.0504
Covered sites 100-1000 bp*	0.3529	0.3465	0.2301	0.3701	0.3365	0.2986	0.4550	0.1068
Covered sites 100-400 bp*	0.5756	0.4721	0.3877	0.6503	0.7259	0.4126	0.6296	0.2439
Covered sites 100-200 bp*	0.5986	0.5589	0.5524	0.7354	0.6872	0.4372	0.6746	0.2884
Fraction of MQ 20 reads aligning to predicted sites	0.8363	0.8090	0.9000	0.9485	0.8373	0.8810	0.8874	0.8147
				**Rice**				
Total predicted sites	371,222	1,486,508	1,037,100	301,435	78,181	60,707	1,204,615	260,304
Predicted sites 100-1000 bp	188,264	875,739	641,398	128,513	14,728	10,684	565,466	111,036
Predicted sites 100-400 bp	90,536	623,512	408,979	70,456	6,146	4,093	371,004	49,065
Predicted sites 100-200 bp	37,870	308,092	183,142	32,571	2,145	1,175	188,652	19,823
Total predicted sites covered*	0.2150	0.5132	0.4420	0.3281	0.1224	0.0867	0.4048	0.1757
Covered sites 100-1000 bp*	0.3578	0.7423	0.6226	0.6715	0.6005	0.4536	0.7235	0.3819
Covered sites 100-400 bp*	0.7026	0.8692	0.8513	0.9136	0.8967	0.6426	0.8732	0.5978
Covered sites 100-200 bp*	0.8202	0.8684	0.8575	0.9062	0.9152	0.7668	0.8606	0.5681
Fraction of MQ 20 reads aligning to predicted sites	0.7138	0.9224	0.9457	0.9854	0.9138	0.9361	0.9484	0.8298

*A read alignment with MQ ≥20 is required for a site to be considered “covered”.

### Effect of fragment size on coverage

**Figure 4:**
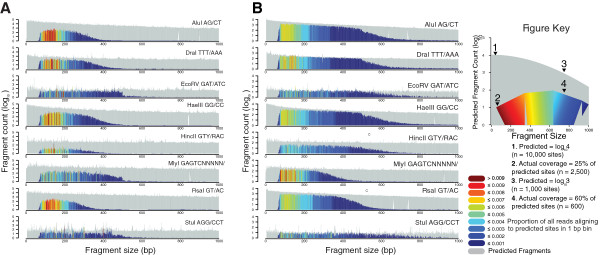
**Size distribution and sequencing coverage of predicted sites.** Sites were predicted based on *in silico* digests of each enzyme against **A)** maize and **B)** rice reference genomes then plotted by log10 site count in 1 bp bins. Predicted site count is displayed in grey. Sites with aligned reads (MQ ≥20) are displayed in heatmap colors as a non-log scale fraction of predicted sites. The heatmap for covered sites represents the total fraction of aligned reads within a given bin.

DNA fragment size is a major factor affecting coverage in both maize and rice. The largest proportion of covered predicted sites in maize (Figure 4A) and rice (Figure 4B) occurs between 100 and 200 bp in all enzymes. For some enzymes, coverage of predicted sites extends outwards to 400 bp or further, but all enzymes show a reduction in the fraction of predicted sites with sequencing coverage after 400 bp. Therefore, the anchoring of reads to restriction sites and the bias in sequenced fragment sizes were two sources for reduced representation in genome coverage in GBS datasets. Further, depth of sequencing coverage per site tends to be higher for smaller sites in both maize (Additional file 1: Figure S1a) and rice (Additional file 1: Figure S1b), with the highest coverage occurring in sites between 100 and 200 bp. Covered, predicted sites >400 bp had the lowest coverage for all enzymes. Sites between 200 bp and 400 bp occupied an intermediate position. This observation suggests that while a complete coverage saturation of all possible sites may require an excessive number of reads, it is possible to achieve near saturation of sites within a limited size-spectrum at much lower depth of coverage.

### GC content of reads

A source of coverage bias may be base composition of fragments due to poor amplification in the PCR step of library preparation. The protocol tried to minimize this bias by keeping the PCR cycles, necessary for indexing, to a minimum. *In silico* predicted sites, based on proximal restriction sites, were used to estimate bias in actual coverage due to the effect of base composition ratios. The GC ratios of all predicted sites between 100 and 200 bp were compared to the GC ratios of actual sequenced reads aligning to predicted sites between 100 and 200 bp in size for all tested enzymes in maize (Additional file 2: Figure S2a) and rice (Additional file 2: Figure S2b). Sites/reads were placed in 2.5% GC-content bins from 0 to 100% and predicted versus sequenced read distributions were compared via two-tailed paired t-test. No bin showed a significant difference (*p* ≤0.05) after correction for false discovery rate [38]. This suggests that the low number of cycles employed in barcoding and amplification (5-6) and the Kapa HiFi PCR reagents likely minimized any PCR bias in AT or GC rich regions.

### Site density

**Figure 5:**
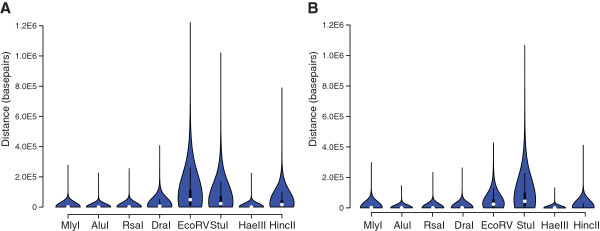
**Distribution of distances in bp between predicted digest sites with sequencing coverage.** Density of sequenced sites is displayed via normalized violin plot for all enzymes based on the distribution of distances between predicted sites with aligned reads for **A)** maize and **B)** rice.

A factor important for the design of GBS experiments is the density of restriction site motifs found in a given genome. Site density will affect the ability to resolve recombination breakpoints and overall number of variants discovered. The distribution of distances between covered predicted sites with sequencing coverage was determined for all enzymes in maize (Figure 5A) and rice (Figure 5B). For all enzymes the shortest distance between predicted sites was 0 bp, indicating both upstream and downstream sequencing from a restriction site. In maize, AluI had the shortest mean distance between covered sites (811.2 bp ±1739.2 bp (*SD*)) followed shortly after by HaeIII (811.8 bp ±1928.8 bp (*SD*)). The longest mean distances between covered sites occurred in EcoRV (79430.0 bp ±91774.1 bp (*SD*)) followed by StuI (48470.0 bp ±66580.2 bp (*SD*)). In rice, AluI had the shortest mean distance between covered sites (251.7 bp ±805.5), followed by HaeIII (516.0bp ±1234.1 bp (*SD*)). StuI had the longest mean distance (70400.0 bp ±84923.3 bp (*SD*)) followed by EcoRV (38600.0 bp ±45027.4 bp (*SD*)). The longest interval without a covered site observed in any organism was 1.2 Mbp (EcoRV, maize).

### Coverage in genic regions

**Figure 6:**
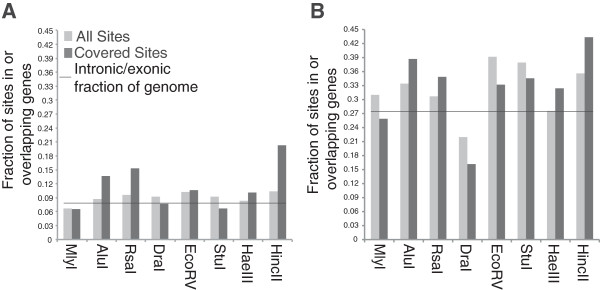
**Fraction of predicted versus covered sites in genic regions.** The fraction of total predicted sites in genic regions was compared to the genic fraction of predicted sites with actual sequencing coverage for **A)** maize and **B)** rice.

Another important parameter in experimental design was the fraction of predicted sites with sequencing coverage in genic regions. Markers in genic regions are generally held to be more informative than non-genic markers as they are less repetitive and, for many studies, more likely to be in proximity of a trait-associated polymorphism. The genic fractions of all predicted sites and sites with sequencing coverage in genic regions were determined (Table 3). Predicted genic site fraction varied from enzyme to enzyme, but in maize (Figure 6A) (Additional file 3: Figure S3a) the covered genic fraction for HincII (0.104 predicted, 0.203 covered), AluI, (0.087 predicted, 0.134 covered) and RsaI (0.095 predicted, 0.153 covered) were considerably higher than predicted. In rice (Figure 6B) (Additional file 3: Figure S3b), covered genic fractions tended to be closer to predicted genic fractions for all enzymes tested. To better understand the ratio of the total predicted and covered predicted genic fractions, termed genic enrichment, the maize and rice genomes were divided into 1 Mbp bins. The predicted versus covered genic ratio was plotted for each of these bins and graphed. While both the predicted and covered genic fractions did vary from bin to bin based, likely based on genic fraction within the bin itself, the relationship between the two was consistent for most enzymes (Additional file 3: Figure S3a, b).

**Table 3:** **Genic fractions of total and covered predicted sites**

	MlyI	AluI	RsaI	DraI	EcoRV	StuI	HaeIII	HincII
				**Maize**				
Fraction total predicted genic sites	0.0668	0.0868	0.0957	0.0921	0.1021	0.0921	0.0836	0.1040
Fraction predicted genic sites 100-1000 bp	0.0621	0.0900	0.0982	0.0766	0.0820	0.0542	0.0823	0.0967
Fraction predicted genic sites 100-400 bp	0.0543	0.0908	0.1011	0.0624	0.0940	0.0428	0.0804	0.0953
Fraction genic sites 100-200 bp	0.0538	0.0900	0.1068	0.0560	0.0780	0.0495	0.0804	0.0865
Fraction total covered sites genic*	0.0657	0.1368	0.1531	0.0768	0.1067	0.0669	0.1007	0.2031
Fraction covered sites genic 100-1000 bp*	0.0660	0.1321	0.1488	0.0757	0.1050	0.0646	0.0967	0.1993
Fraction covered sites genic 100-400 bp*	0.0638	0.1317	0.1484	0.0742	0.1093	0.0664	0.0964	0.1958
Fraction covered sites genic 100-200 bp*	0.0630	0.1321	0.1503	0.0661	0.0928	0.0763	0.0961	0.1737
				**Rice**				
Fraction total predicted genic sites	0.3102	0.3342	0.3070	0.2196	0.3917	0.3794	0.2756	0.3561
Fraction predicted genic sites 100-1000 bp	0.2793	0.3367	0.3261	0.1721	0.3124	0.2261	0.2977	0.3021
Fraction predicted genic sites 100-400 bp	0.2471	0.3442	0.3112	0.1439	0.2790	0.2084	0.2910	0.3021
Fraction genic sites 100-200 bp	0.2244	0.3468	0.3041	0.1328	0.3016	0.2417	0.2929	0.2867
Fraction total covered sites genic*	0.2588	0.3871	0.3487	0.1619	0.3321	0.3455	0.3240	0.4336
Fraction covered sites genic 100-1000 bp*	0.2625	0.3837	0.3502	0.1646	0.3334	0.3413	0.3222	0.4178
Fraction covered sites genic 100-400 bp*	0.2587	0.3796	0.3435	0.1504	0.2970	0.2958	0.3180	0.4178
Fraction covered sites genic 100-200 bp*	0.2423	0.3825	0.3366	0.1395	0.3158	0.3019	0.3241	0.4060

*A read alignment with MQ ≥20 is required for a site to be considered “covered”.

### Enzyme methylation sensitivity

One possible factor responsible for the enrichment of covered sites in genic regions relative to the predicted values for some enzymes is cytosine methylation sensitivity of the restriction enzyme. Repetitive DNA in plants tends to be methylated at CpG and CpNpG motifs. Digestion of repetitive gDNA by methylation sensitive enzymes may result in DNA fragments too large to sequence being generated, whereas non-methylated regions would produce a normal DNA size spectrum.

To assess the contribution of cytosine methylation to genic enrichment, the nucleotide ratios flanking the motifs of restriction sites were compared in predicted sites with aligned reads for a given enzyme versus the total set of predicted sites. Sites were further broken up into ones overlapping introns and exons and sites in non-genic regions, as repetitive, intergenic regions are often methylated. This analysis indicated that in maize several enzymes, namely HincII, RsaI, and AluI show considerable reductions in guanine one to two bp upstream and cytosine one to two bp downstream of restriction motifs. Further, this difference is more pronounced in non-genic than in genic regions (Additional file 4: Figure S4a).

In maize, HincII was sensitive to both CpNpG and CpG methylation. HincII had the largest genome-wide decrease between predicted and covered upstream cytosine (from 0.227 to 0.123) and downstream guanine (from 0.225 to 0.128) ratios. Further, it had greatest increase in covered versus predicted sites in genic regions of all tested enzymes (10.04% sites predicted to be in genes, 20.03% covered sites in genes, 1.95-fold increase). RsaI, showed clear sensitivity to CpG methylation but was much less sensitive to CpNpG methylation. RsaI showed a 1.45-fold enrichment in predicted sites with sequencing coverage in genic regions versus all predicted sites (9.57% predicted, 15.31% actual). Interestingly, the enzyme with the third highest increase covered genic fraction relative to predicted (1.58-fold) was AluI, which, due to its recognition motif of AGCT, was only sensitive to CpNpG methylation.

In the less repetitive rice genome, predicted versus covered nucleotide ratios were similar for most enzymes, and differences between covered and predicted sites for a given enzyme in rice were smaller than in maize (Additional file 4: Figure S4b). In rice, HincII was the enzyme with the largest difference in G/C ratios between predicted and covered sites. The cytosine ratio 1 bp upstream of the HincII motif decreased from 0.240 to 0.189 and the guanine ratio downstream decreased from 0.239 to 0.192 between total and covered predicted sites. That G/C ratios would be closer between covered and predicted sites in rice than maize was expected, as no enzyme in rice had a covered sites genic fraction >25% that of predicted sites. These results indicated that benefits conferred from methylation sensitive enzymes are genome dependent.

It is worth noting that, while different enzymes showed different degrees of methylation sensitivity in this study, this may be a product of the genomes tested more than an intrinsic property of the enzymes themselves. If an enzyme’s recognition motif predisposes it to cut more often in a repetitive region, it may appear more methylation sensitive than one whose recognition site biases it away from these regions.

### GBS-based population genomics

**Figure 7:**
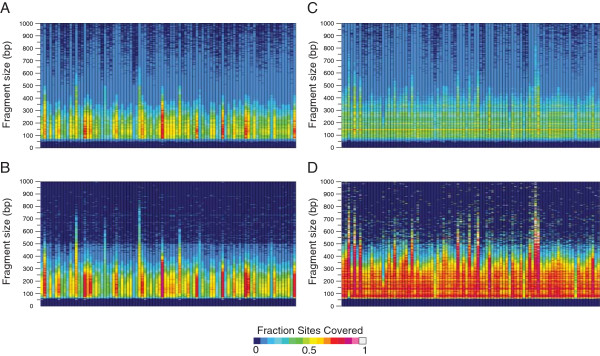
**Fraction of predicted sites covered in samples from a F**_**2**_ **admixture population.** Reads from each F_2_ sample were aligned to predicted sites, then predicted sites were placed in 2 bp bins, with the fraction covered in each bin indicated by the heatmap. **A)** The RsaI dataset, aligned against total predicted sites. **B)** RsaI dataset, aligned against the subset of predicted sites with sequencing coverage in the original RsaI B73 GBS experiment. **C)** HincII dataset, aligned against total predicted sites. **D)** HincII dataset, aligned against predicted sites with at least one read coverage in the original HincII experiment. Sample order is given, left to right, in Additional file 5: Table S1.

The low cost and high multiplexing capacity of the modified GBS protocols indicated that the method would be suitable for population genomics. To test the suitability for trait mapping and population structure analysis, RsaI and HincII restriction digestions were used to create multiplexed GBS libraries from an F_2_ population (*n* =91) derived from a cross between B73 and Country Gentleman (CG) maize inbreds. Eighty-nine RsaI samples and ninety HincII samples were analyzed, with eighty-eight in common to both libraries along with both parental inbreds (Additional file 5: Table S1). Reads were demultiplexed and aligned to all predicted and covered sites in the B73 reference datasets for RsaI (Figure 7A, B) and HincII (Figure 7C, D). No evidence was found of bias due to barcodes, as regression analysis found little correlation between samples sequenced with the same barcodes between HincII and RsaI (slope =0.087, *r*^2^ = 0.071), excluding the fourteen HincII samples that were resequenced. There remains the possibility that certain, specific barcodes will underperform, but these are likely to be only identified through repeated experiments.

As with previous experiments, the results indicated that the highest fraction of covered sites was between 100 and 400 bp. In this range, F_2_ sites were more concordant with predicted sites covered in the reference B73 datasets as expected. Above 500 bp, the performance of the set of predicted sites covered by the B73 HincII and RsaI datasets was no better than the total set of predicted sites for most F_2_ samples (Figure 7A-D).

### Variant calling and imputation

**Figure 8:**
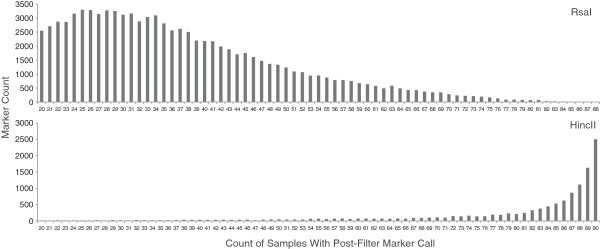
**Number of samples with a call passing quality control filters for each marker in final B73xCG F**_**2**_ **datasets.**

Variant filtering is a critical step in identifying informative markers, and special methods are required for GBS datasets. Variants were filtered using a combination of standard and population genomics based criteria. Filtered variants were required to be homozygous, opposite calls in parentals, covered at 2× or greater in at least twenty F_2_ individuals, MQ and Phred score >30, and *r*^2^ correlation ≥0.3 with five proximal variants upstream or downstream. A total of 12,499 post-filtration variants were identified in the HincII dataset and 91,894 post-filtration variants were identified in the RsaI dataset (Additional file 5: Table S1). For the RsaI there was a mean per-sample post-filter variant count of 38,439.1 ± 22,133.1 (*SD*) (Figure 8A), while HincII had a mean per-sample post-filter variant count of 11,214.7 ± 1093.4 (*SD*) (Figure 8B).

**Figure 9:**
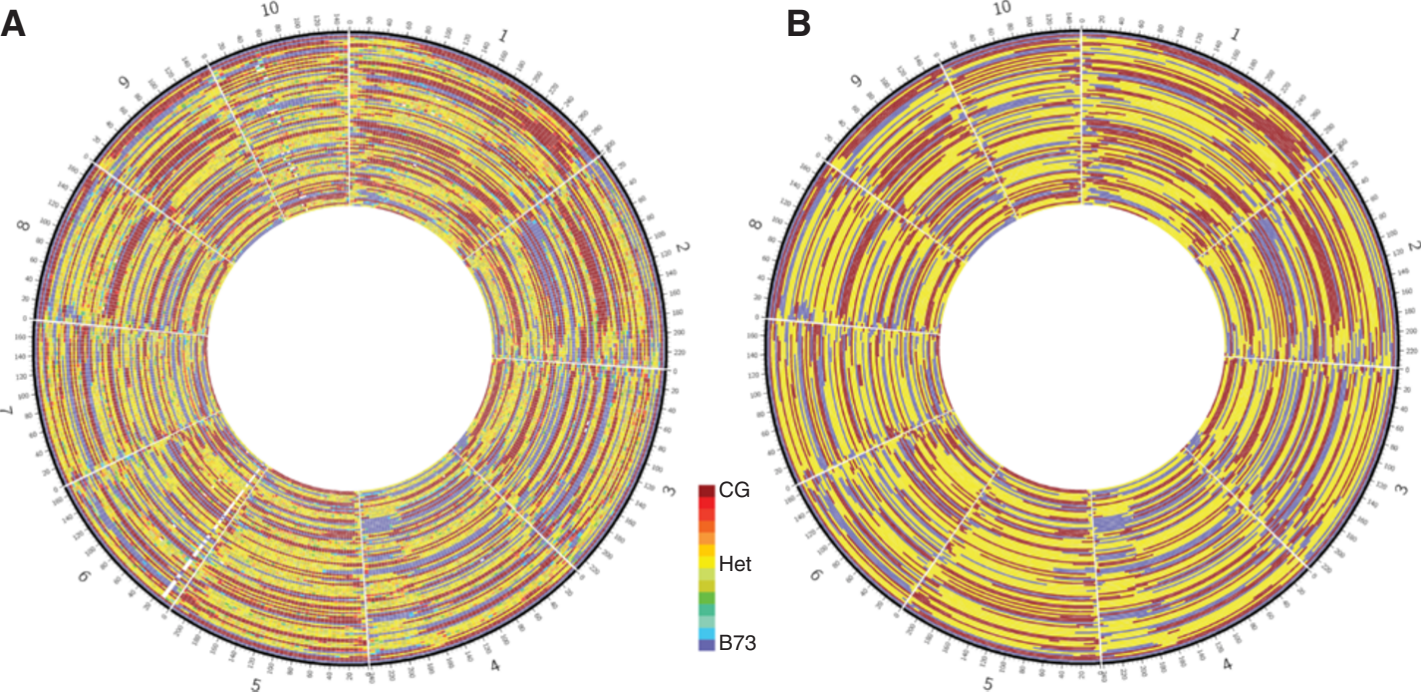
**Imputed RsaI GBS datasets from an F**_**2**_ **admixture population. A)** Phased, filtered variants displayed by “mean” genotype as indicated by heatmap in 5 Mbp bins. **B)** Post imputation genotypes. Sample order is given, outermost to innermost, in Additional file 5: Table S1.

Next, parental contribution and recombination breakpoints were determined by imputation of variants by first phasing the final set of variants by parental genotype (Figure 9A, Additional file 6: Figure S5a, Figure S5b) then applying a least squares algorithm with a sliding window for final genotype calls (Figure 9B, Additional file 7: Figure S6a, Figure S6b). F_2_ samples typed in both the HincII and RsaI datasets had a concordance of 97.89% ±1.00% (*SD*) on a genomewide, nucleotide level. While large regions with a single genotype were consistent with some minor variation in imputed breakpoint position, the genotype of smaller regions varied between some replicates of samples covered in both the HincII and RsaI datasets (Additional file 8: Figure S7). These differences may be due to reduced per-variant sequencing coverage in the RsaI dataset resulting in false homozygous calls in heterozygous regions, or reduced marker density in the HincII dataset resulting in events being missed.

### Trait mapping

**Figure 10:**
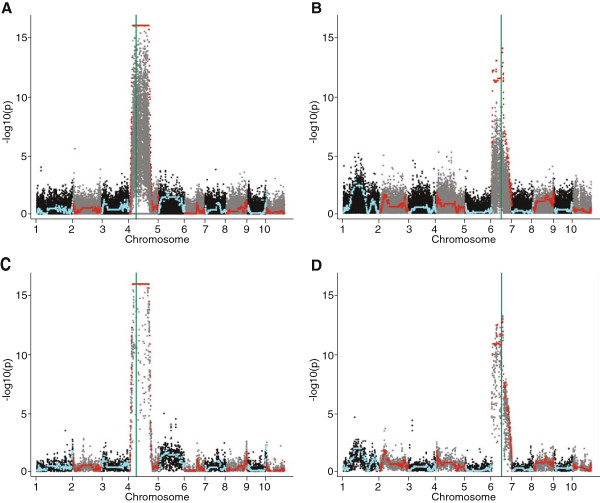
**Trait mapping for** ***yellowy*** **(** ***y1*** **) and** ***sugary*** **(** ***su1*** **) in an F**_**2**_ **admixture population.** A green line in the plots annotates the locations of both genes. Pre-imputation markers are shown in black and grey. Markers, post-imputation and error correction are shown in color. **A)** RsaI GBS dataset, *su1* map. **B)** RsaI GBS dataset, *y1* map. **C)** HincII GBS dataset, *su1* map. **D)** RsaI GBS dataset, *y1* map.

The F_2_ population segregated for two recessive traits previously mapped in maize: *sugary1* (*su1)* and *yellowy1* (*y1)*. The *su1* gene maps between Chr4: 41,369,510-41,378,299, and *y1* maps between Chr6: 82,017,148-82-020,879. To further validate our variant calling and imputation efficacy of our GBS methodology, these traits were mapped using GBS in the F_2_ population. A one way ANOVA test on both pre and post imputation datasets of post-filter markers (Figure 10A-D) were able to localize causative alleles in the correct regions with *p* <1E-10.

### Coverage simulations

**Figure 11:**
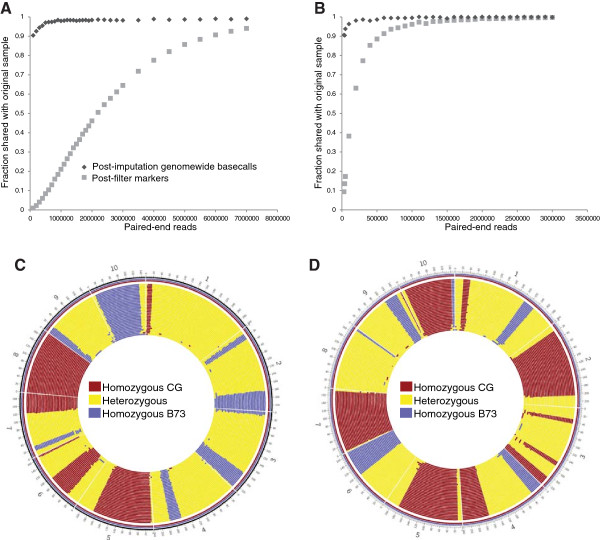
**Effect of read count on marker dataset size and imputation.** Fraction of shared post-filter, pre-imputation genetic markers and fraction of post-imputation shared genome with original sample for subsamplings of **A)** RsaI F_2_-44 and **B)** HincII F_2_-23. **C)** Imputed genomes for each subsample in RsaI F_2_-44 displayed in concentric rings. Sample read count declines from the outermost ring to the innermost. **D)** Imputed genomes for each subsample in HincII F_2_-23 displayed in concentric circles. Sample read count declines from the outermost ring to the innermost.

An important consideration in multiplexing for population studies is the per sample depth of coverage. To determine how depth of sequencing coverage affected imputation and marker calling, multiple subsets of randomly selected reads were taken from one RsaI F_2_ sample (F_2_-44) and one HincII F_2_ sample (F_2_-23). These samples were selected due to their high read-count, which resulted in a near saturation of potential markers (91,584 of 91,894 and 12,154 of 12,499, respectively). Subsets were then realigned against the reference genome, variants were called, and genotypes were imputed. The original RsaI sample contained 15,398,878 reads and 75,593 variant calls. To obtain 90% of the original sample’s variant calls, 5,500,000 reads were required (Figure 11A). The original HincII sample contained 3,698,544 reads and 9728 variant calls. Results indicated that as few as 550,000 reads were required to obtain 90% of the imputed variant calls found in the primary sample. (Figure 11B). The post-imputation genome similarity with the original sample remained above 90% in all read subsets. In both the post-imputation RsaI (Figure 11C) and HincII (Figure 11D) datasets, as the number of reads decreased, small recombination events disappeared and possible artifacts began to appear. For RsaI, imputed genome similarity, as measured against the original, high-coverage sample fell beneath 98.0% at 800,000 reads while genome similarity at 100,000 reads fell to only 90.4%. Discordant recombination breakpoints, defined as a pattern of recombination different from that of the primary sample, began to appear at 1.6 million reads. These incongruities were seen as minor segments of miscalled genotypes and discordant localization of recombination breakpoints. For HincII, genome similarity remained at 98% at 100,000 reads and the lowest percent genome similarity was 90.4% at 40,000 reads. Discordant recombination breakpoints began to appear at 500,000 reads.

## Discussion

Genotyping-by-sequencing is a high-throughput, low-cost technology that meets the need for robust variant identification in diverse populations from a variety of species [18, 39–43]. The extant GBS technology has several limitations that were addressed in this study. The use of enzyme-specific barcoded adaptors means that for each utilized enzyme a number of doubled-stranded adaptors equal to multiplexing targets must be developed. This results in a high cost to initially implement GBS and to switch enzymes, discouraging changes in experimental design even when an alternative enzyme may better meet experimental needs.

### Modifications to GBS

To improve both flexibility and scalability of GBS we modified the original protocol in a number of ways. The most important and novel change was to remove the requirement for custom enzyme-specific barcoded adaptors. To make this change, restriction enzymes were chosen that created blunt-end fragments that required a single adenylation step for compatibility with standard Illumina Y-adaptors. Next, DNA barcodes required for multiplexing samples were added to the universal adaptors during a low-cycle PCR step. This dual indexing system allows a great number of samples to be multiplexed during sequencing to minimize cost. For instance, with just twenty indexed forward and twenty indexed reverse primers as many as four hundred samples can be multiplexed on a single HiSeq 2500 lane. Finally, a bead-based in-solution library preparation protocol facilitates automation and allows for gel-free size selection.

Over forty blunt-end enzymes compatible with this GBS protocol are commercially available. We selected eight enzymes that represented a variety of recognition motif lengths, sequence contents, and methylation sensitivities to test the robustness of these new methods. This panel of enzymes was used to create GBS datasets from two reference genomes *Z. mays* B73 [35] and *Oryza sativa japonica* Nipponbare [36]. Haploid genome length (approximately 2500 Mbp and 430 Mbp respectively), repeat content, methylation, and genic fraction differ considerably between the two genomes. In addition, a maize F_2_ population consisting of ninety-one individuals was created from two maize inbreds B73 x Country Gentleman and genotyped by GBS using two enzymes, RsaI and HincII.

### Prediction of coverage

The vast majority of reads for all enzymes align to proximal restriction sites. Further, these sites tend to be between 100 bp and 400 bp in size (Figure 3, Additional file 1: Figure S1). This is likely a result of the size selection step during the library preparation and the bias of the Illumina sequencer towards smaller fragments.

Mispair sites tended to have lower coverage than predicted sites across all enzymes, but their >1× coverage values indicated that some mispair events were reproducibly covered. These may have been generated as a result of polymorphism or methylation disrupting a restriction site or the digest of a given site inactivating proximal ones.

Singlet sites, events where one end of a read aligned to a restriction site and the other end aligned to random DNA could also be generated from two potential sources. The first possibility is a polymorphism creating a restriction site that was not found in the reference. The other possibility is that the restriction site occurs near the random end of a DNA fragment. The later is the most common case, as in most samples singlet sites were at or barely above 1× mean coverage, which suggests singular events.

Null sites occurred when neither end of a read aligned to a restriction site. For MlyI, DraI, HincII, EcoRV, and StuI in maize and all enzymes in rice save MlyI, these sites had a mean coverage near 1×, suggesting they were the result of random DNA fragments being sequenced. In AluI, HaeIII, and RsaI in maize, coverage was considerably above 1×, though the number of unique sites was small compared to the others. The likely reason for this is that some reads were misaligned to the same location in the genome multiple times. Several observations support this. First, as random fragments are generated from degradation, a consistent amount of these would be expected to be generated for each library as the amount of input DNA was equal between them. For enzymes that cut rarely and produce relatively few reads, such as DraI, HincII, EcoRV, and StuI, these would make up a larger overall proportion or the reads than for enzymes that cut frequently and generate large numbers of potential reads, such as AluI and HaeII. Second, misaligned reads represent a fraction of the total amount of reads generated and aligned. Thus, high coverage null sites are observed for enzymes that cut frequently and generate large datasets, such as AluI and HaeIII. Finally, null sites with high coverage generated by misalignments would be expected to be more common in maize, due to the highly repetitive and difficult nature of the genome, than in rice, which is much simpler to align reads to. This is also concordant with observations.

Finally, it is worth noting that all reads that are accurately aligned and whose alignments are observed across multiple samples in a population contribute to the value of a dataset, not just reads aligning to predicted sites. Mispair sites are the most common example of this, though singlet sites contribute as well. Given the likelihood that many null sites represent misalignments or broken DNA fragments, however, it may be advisable to filter these reads.

To further examine how well we could predict GBS sequencing coverage, we realigned reads from two datasets, one produced by RsaI and the other by HincII, generated from a B73 × CG F_2_ population to the total set of predicted sites and to the set of predicted sites with sequencing coverage (Figure 7). As was expected from our original datasets, the majority of coverage occurred between 100 and 400 bp. Predictability of coverage, as measured by the fraction of sites covered, improved when an F_2_ sample’s reads were aligned against sites covered in the pilot B73 experiment rather than the total set of predicted sites. In RsaI, this improvement was modest, with many samples only improving 5-10%. In HincII, however, the improvement was considerable. While only 30-40% of the total predicted sites were covered in each F_2_ sample, up to 80-90% of the pilot-experiment sites were covered in the same F_2_ samples. The reasons for this are likely twofold. First, our identification of total predicted sites did not take into account the ability to unambiguously align reads to these sites. The use of a dataset based on predicted sites with sequencing coverage intrinsically did, as there was a MQ ≥20 cutoff for sites. Second, the use of predicted sites with sequencing coverage by nature accounted for sites that were made inaccessible by methylation. The improvement in HincII data quality between the total and covered sites was likely due to this, as HincII is highly sensitive to methylation. Finally, though not as applicable in this case, pilot experiments account for differences between the target genome and the reference genome that cannot be identified *in silico.*

### Enzyme parameters and data quality

Our results clearly show that the ability to use a panel of enzymes for GBS has several clear benefits. A major source of data loss in sequencing is the inability to uniquely align reads with sufficient confidence [37]. As assessed by mapping quality, certain enzymes, such as DraI, StuI, and HincII produced datasets that were aligned with greater accuracy than others, such as MlyI, HaeIII, and EcoRV in maize (Figure 2). This may reflect a bias against repetitive elements due to motif, or it could be methylation sensitivity limiting digest in repetitive regions.

Enrichment of genic regions was another parameter looked at closely. HincII, RsaI, and AluI in maize produced datasets that contained a considerably greater portion of covered sites in genic regions (Figure 6A). On the other hand, for MlyI, DraI, EcoRV, and HaeIII in maize as well as all enzymes in rice (Figure 6B), the proportion of covered sites overlapping genic regions was similar to the genic fraction of total predicted sites. The difference between the two categories appears to be due to methylation sensitivity, which biases enzymes away from cutting the genome in repetitive, heterochromatic regions. The ability to enrich for genic coverage is beneficial in any dataset but is especially beneficial for association studies in populations that have undergone large amounts of recombination. In these studies, a trait may only have associations to markers in the immediate vicinity of the functional variant.

### Restriction motif presence and nucleotide complexity

One initial concern was that the lack of enzyme-specific adaptors might produce more random reads derived from broken DNA fragments. By omitting the end-repair step, we attempted to enrich for digest fragments, as end-repair both fixes broken ends and adds a phosphate group necessary for adaptor ligation to the 5′ ends of the fragment. The phosphate group is naturally retained on the 5′ with a restriction digest [44]. All enzymes save MlyI reliably produced DNA fragments with more than 80% of ends containing the proper restriction motif (Table 1). MlyI, due to its offset cut site, had a restriction motif present in less than half of its reads. Counterintuitive to expectations, this may be beneficial. This is due to how the Illumina software must calibrate both to identify the cluster boundaries on the flow cell and to assess the quality of nucleotide calls. Proper calibration requires that both the red laser, recognizing G/T and the green laser, recognizing A/C, be sufficiently excited, which requires nucleotide complexity at every cycle in the sequencing run. This is especially important in the early cycles [45]. As the restriction site for enzymes recognizing palindromic motifs occur at the beginning of a read, this has the potential to severely disrupt a sequencing run.

For most enzymes, namely ones that cut in the center of a palindromic sequence, this means that approximately 20-30% of a run must consist of a “calibration” sample with a random sequence. When whole genome sequence is desired or the sequencing center can arrange to conduct multiple experiments on a single lane, waste is not an issue due to this. When a full lane is desired, custom sequencing protocols may be used that defer cluster coordinate mapping past the motif-containing sequencing cycles [45] or utilize custom sequencing primers that “mask” the restriction site may be used to avoid low-complexity issues. Further, MlyI and other blunt-end restriction enzymes without a cutsite in the center of a palindromic sequence (for example, Type IIS enzymes) do not have this calibration requirement as half or more of the reads will not contain a restriction motif at all.

### Sequencing efficiency

Overall sequencing efficiency is a point of interest. GBS libraries prepared using this method lack complexity during the initial few cycles of a sequencing reaction, which much be compensated for as discussed above. They also have a considerably wider size range than a randomly sheared library. Regarding the amount of sequencing that can be expected per lane of the HiSeq 2500, we have obtained similar results to standard whole genome sequencing on some libraries. The rice enzyme panel produced just over two hundred million 2 × 75 bp paired-end reads when run on an Illumina HiSeq 2500 (rapid mode) lane, which was approximately 33% above what would be expected from a lane of WGS sequencing per Illumina literature. The maize enzyme panel produced just over one hundred and fifty million reads, or approximately what was expected. The B73 x CG F_2_ populations, both HincII and RsaI, were not run on a single lane however. Both were initially run on 80% of a HiSeq 2500 lane then small amounts of additional resequencing were performed. In the case of RsaI, this was targeted across all samples, whereas for HincII, fourteen specific samples were resequenced. This is likely part of the reason why the coefficient of variation in readcount was much smaller for HincII (0.595) than RsaI (0.928). Variations in sample read count were most likely due to the use of manual pipetting as well as variation in DNA input quantity, as we found no evidence of a correlation between readcounts for samples that shared the same barcodes between datasets (slope = 0.087, *r*^2^ = 0.071). We have found based on later GBS experiments that improvements in normalizing DNA input as well as a switch to automatic pipette systems have reduced sample variation considerably.

### Effect of genome on enzyme selection

Enzyme panels were tested on both B73 maize and Nipponbare rice. While both are critical crop species, their genomes are very dissimilar. The maize genome is large at 2500 Mbp and highly enriched for methylated transposon content. Estimates place the total transposable element content of the B73 genome at above 80% [46]. The rice genome is much smaller at approximately 430 Mbp and is much less repetitive at approximately 40% [47]. These parameters resulted in very different experimental outcomes.

The first, and most obvious difference was in the fraction of reads that could be aligned to a genome with high confidence, represented by mapping quality. On average, twenty percent more reads could be aligned with a MQ ≥30 in rice than in maize (Figure 1). This is not an unexpected result. What was unexpected was that while paired-end reads conferred a statistically significant improvement in alignment quality over single end reads in maize, they did not do so in rice. In fact, the opposite was observed. Again, this is likely due to the differences in repetitive content between the two genomes. Additional sequence was able to improve the rate of alignment in maize, but in rice, where shorter sequences were more likely to be suitable for a unique alignment, additional sequence just increased the likelihood of sequencing errors reducing the alignment quality.

The second experimental outcome that differed greatly between the two genomes was methylation sensitivity. In maize, HincII, RsaI, and AluI showed significant reductions in G/C content surrounding the restriction motif at sequenced sites versus the predicted G/C content of all possible sites (Additional file 4: Figure S4a). Further, the fraction of covered reads in genic regions was also greater than predicted by as much as twofold (Figure 6A). In rice, the proportion of covered sites in genic regions was higher than in maize, the differences between the total predicted and covered datasets tended to be much smaller (Figure 6B). Further, there was little or no evidence of bias against restriction sites with a potentially methylated motif for any enzyme (Additional file 4: Figure S4b). This follows the observation that the maize genome contains a much larger proportion of methylated, repetitive content than rice.

The conclusion of the genome comparison, that enzyme choice should take into account the genome of the target organism is not surprising. Utilization of methylation sensitive enzymes avoids repeat content in methylated, repeat rich genomes. Paired-end sequencing in difficult, highly repetitive genomes may produce a considerable increase in useable markers, whereas in much simpler genomes the use of single-end sequencing this may not be an issue. One area that was not directly examined in this study but would likely improve data quality is the use of restriction enzymes that are biased away from repetitive regions by the sequence of their recognition motif. Identifying transposon families or repetitive elements likely to be present in a given genome and selecting an enzyme that does not recognize their sequence may further reduce coverage of unformative regions.

### Variant calling and filtering

GBS datasets present unique challenges to variant calling and filtering. While traditional metrics like mapping quality and Phred score can be applied, the fixed ends of GBS fragments confound the allelic balance metric and the removal of PCR duplicates by collapsing non-unique reads. Incorporating a low cycle PCR step minimized the latter issue but GBS variant filtering required additional metrics, such as linkage disequilibrium, heterozygosity, and Hardy-Weinberg Equilibrium (HWE). Each of these metrics has circumstantial utility. For instance, linkage disequilibrium analysis requires a reference genome with contigs or scaffolds of sufficient size to compare markers. In wild populations, linkage disequilibrium is highly dependent on population history [48]. HWE is a useful metric for wild populations, but artificial crosses may have issues with segregation distortion or non-random mating. Heterozygosity is applicable to many experiments, but measurements should be corrected for coverage and take into account population history. A final note for any error correction is that variants called from paired-end reads aligning to the same position should be collapsed to a single datapoint when attempting admixture analysis or trait mapping and should be weighted accordingly. When treating paired-reads as single-end tags, this may cause allelic bias if each tag is treated independently. Many of the error correction tools and concepts have been built into TASSEL, a software package developed for GBS analysis [29].

### Trait mapping in an F_2_ population

To test our modified GBS protocol, we mapped two traits, *yellowy (y1)* and *sugary (su1)* in a maize F_2_ population of ninety-one individuals. Correct locations for each causative allele were identified with both tested enzymes, RsaI and HincII. While data imputation did confer additional significance to association measurements, filtered, unimputed markers were still able to correctly identify the regions containing the causative alleles (Figure 10).

RsaI, as was suggested by its marker density profile and overall less complex motif, was able to identify over ninety thousand post-filter markers, compared to just over twelve thousand post-filter markers in the HincII dataset (Additional file 5: Table S1). In addition, each RsaI sample had, on average, three times as many covered markers as per HincII sample. The RsaI and HincII samples both underwent approximately the same amount of sequencing. At first glance, this indicates RsaI was the better enzyme. Higher marker density leads to better resolution of recombination breakpoints. However, what is also noteworthy is the number of samples covered per marker (Figure 8). With HincII, markers were covered across almost every sample, while in RsaI each marker was covered in only ~30% of samples. Further, many RsaI markers even within a few cM to the mapped locations of *y1* and *su1* did not necessarily show significant association with their respective phenotypes pre-imputation. In HincII, virtually every marker surrounding the previously identified locations for the two mapped traits showed a significant association with phenotypes pre and post imputation. Thus, in scenarios where imputation is not possible, enzymes with a long, complex motif resulting in a more limited set of covered sites may be desirable.

## Conclusions

Next generation sequencing has clearly demonstrated its utility for generating large, robust datasets for population genomics in humans. Migrating these methods and utilities to other reference organisms has been met with difficulty, however. The major obstacle has traditionally been poor or non-existent reference genomes combined with the high cost of developing oligo capture arrays required for exome sequencing, the most popular method for genotyping in humans. Nonetheless, low-cost, highly scalable sequencing is a critical requirement for large-scale population genomics in any species. Since the introduction of RAD sequencing seven years ago, methods have steadily progressed that answer this need. Our modified GBS protocol represents another step that significantly improves on pre-existing capacity while adding several new ones.

Most critical amongst these refinements is the low starting cost adaptors, primers and reagents required for implementation. Compatibility with numerous blunt-end restriction enzymes allows for enzyme parameters to be matched to the needs of a given experiment. Further, the switch to Illumina Y-adaptors results in reduced concatamer formation due to the dA tailing step, which in turn improves the quality of paired-end sequencing data.

The resulting protocol has several advantages and disadvantages compared to the original protocol described by Elshire et al. The key advantage is the ability to switch between restriction enzymes with no change in utilized primers or adaptors. Further, compatibility with Illumina Y-adaptors, paired with the dA tailing step, prevents concatamer formation, increases the sequenceable fraction of the library, and allows for paired-end sequencing. Finally, the use of Illumina Y-adaptors allows the PCR incorporation of dual-indexed barcodes during library amplification, which facilitates large-scale, inexpensive multiplexing.

There are several disadvantages, however, compared to the Elshire et al. protocol. First, the use of custom adaptors allows for modulation of the barcode length, whereas this protocol requires the “spike-in” of approximately 20% random DNA to a sequencing lane to prevent sequencer calibration problems from arising due to reduced nucleotide complexity. This can be avoided by the use of custom sequencing primers that mask the restriction motif or the use of “dark cycling”, which is the continuation of the non-imaging portion of the sequencing PCR reaction through invariant bases. In addition, the use of custom adaptors specific to an enzyme overhang reduces the number of sequencing reads originating from random, broken DNA fragments. These random, broken ends occur on less than 1% of the sequencing reads for enzymes such as AluI, but may occur in up to or over 10% of the reads in HincII, StuI, and EcoRV.

The key factors that must be balanced in any GBS experiment are multiplexing, resolution, and coverage. Optimal marker density for QTL mapping and other population genomics increases with the expected number of recombination events per sample and sample size. This can be empirically calculated to a degree [49]. All three are directly affected by enzyme choice. A four base pair cutting enzyme will produce a dense site profile across the genome but large amount of sequencing is required to obtain coverage on predicted sites. A six base pair cutting enzyme will produce a sparse site profile, but less sequencing will accomplish coverage saturation. As demonstrated by our B73 × CG F_2_ population, even a simple imputation method resolved these issues by removing ambiguous data. However, imputation remains a critical area for improvement in GBS.

Many popular imputation algorithms are designed specifically for human data [50]. These methods often assume high per-marker accuracy, complex haplotype, and the availability of a reference genome. GBS datasets, on the other hand, may have significant amounts of missing or inaccurate data. Haplotypes may be complex in some cases, but in many experiments parental data will be available and genotypes can be phased in a straightforward manner. Reference genomes are often not available or are incomplete. While popular methods such as fastPhase can be applied to GBS data [51–53], pre-processing is advisable. Pre-processing should test for false homozygotes resulting from low coverage and collapse non-independent markers into single values. Non-independent markers are polymorphisms called from a set of reads aligned to the same location, which is typical with GBS experiments. Errors, including misalignment, false homozygosity, and paralogous sequence will be common to all markers originating from this set of reads. Improperly accounted for, they may offer multiple, seemingly independent confirmations of a false genotype that may produce an incorrect result from imputation. Thus, it is recommended that all markers from the same set of reads be treated as a single event rather than independently.

In the case of datasets from organisms with non-existent or incomplete reference genomes, namely ones that exist as unscaffolded contigs, algorithms designed for humans fail entirely. Imputation methods do exist that are suitable for these datasets that can provide high levels of accuracy [54, 55]. While differing in implementation, these methods consistently rely on identifying proximal markers through linkage disequilibrium. As such, an initial dataset with only a modest number of missing markers is advisable when employing these methods. In addition, data with a high error rate may be unsuitable for these algorithms.

Imputation methods designed for GBS are implemented to incorporate parental data into phasing and, when necessary, impute missing parental genotypes from population data. Further, they do not assume Hardy-Weinberg equilibrium or random mating, as may be the case with many populations. Many, however, are designed to work with NAMs or other populations without heterozygosity [30, 31, 56, 57]. Of the GBS capable imputation methods that do exist, most are designed for inbred lines where heterozygosity is largely absent. For populations with large remaining amounts of heterozygosity, these methods are unsuitable. Thus, the next critical field for improvement in GBS is likely to be an imputation algorithm or package of algorithms that can answer its unique requirements.

The choice of enzyme is therefore highly dependent on available data resources. In a population with a well-established reference genome and little heterozygosity, imputation may reconcile a dataset with large amounts of missing markers into a robust genetic map. In an organism with a contig-level or non-existent reference genome, selecting an enzyme with a sparse profile so each marker is covered in a large number of samples may be desirable. That being said, most error correction methods will require that a given marker have sequencing coverage across a sufficient number of samples.

GBS has already demonstrated viability in trait mapping, admixture analysis, genome wide association, population genomics, and characterization of diversity in reference and non-reference organisms [58]. The modifications described here increase the portability of GBS to individual labs interested in adopting it by reducing the initial cost of oligos, allowing for simple, low-cost, pilot experiments, and integrating library preparation more directly into the standard Illumina pipeline.

## Methods

### GBS library preparation and sequencing

Leaf tissue was collected from the rice Nipponbare, maize inbreds B73 and Country Gentleman, the B73xCG F_1_ hybrid and 91 of its F_2_ progeny. DNA was extracted from leaf tissue as described [59]. Approximately 500 ng of genomic DNA per sample was hybridized onto AMPure XL SPRI beads (AG3880, Beckman Coulter), cleaned as described in Broad Institute Protocol [34], and digested with a 5-fold excess of restriction enzymes under manufacturer specified conditions for 2 hours. Genomic DNA from B73 and the Nipponbarre was digested with MlyI (R0610), AluI (R0137), RsaI (R0167), EcoRV (R0195), StuI (R0187), HaeIII (R0108), and HincII (R0103, New England Biolabs). For the F_2_ mapping population, RsaI and HincII were used to digest genomic DNA. Of the ninety-one F_2_ individuals in the B73 × CG mapping population, eighty-nine were processed with RsaI, and ninety were processed with HincII.

Following digestion, a modified version of the standard Illumina library preparation was performed. The first modification was the omission of the end-repair step. As restriction enzymes compatible with this protocol produce blunt-end, 5′ phosphorylated DNA fragments, end-repair is unnecessary. Further, end-repair would fix random, broken DNA fragments and add phosphate groups to their 5′ ends. This is undesirable as these ends would be highly random and result in irreproducible noise being added to the dataset. The second modification is the replacement of column-based cleanup with a Solid Phase Reversible Immobilization (SPRI) bead based methodology [33] as implemented by the Broad Institute [34]. In this method, double stranded DNA is immobilized on the paramagnetic beads held in place during buffer exchange, DNA size selection and cleanup steps. Wash, elution, and hybridization buffers were as described in the Broad Institute protocol. Following addition of beads, they are retained throughout the protocol until the post-adaptor ligation size selection step.

### dA tailing and adaptor ligation

Following digestion, samples were immobilized to the SPRI beads via addition of well-mixed beads at 3× concentration, then a wash was performed as described in the Broad Institute protocol. The end-repair was omitted for the reasons described above and dA tailing was performed. The addition of a 3′ adenine to DNA fragments ensures compatibility with standard adaptors while preventing concatamer formation. For dA tailing, samples were first eluted with 40 μL of 10mM Tris-HCl, then dA tailing was done with Klenow Fragment (3′-5′ exo-) (M0212, New England Biolabs) per manufacturer’s instructions.

Following dA tailing, samples were once again washed per Broad Institute protocol. After elution into 40 μL of 10 mM Tris-HCl, Illumina Y-adaptors (Additional file 9: Table S2) were ligated to DNA fragments using standard Illumina protocol with Broad Institute modifications for SPRI based library preparation [34]. Ligation is done using the Quick T4 DNA ligase kit (M0202M, New England Biolabs) per manufacturer’s protocol.

### SPRI-based size selection

A key advantage of SPRI based DNA manipulation is the ability to perform gel-free, in-solution size selection of DNA fragments. By varying the concentration of polyethylene glycol (PEG) in the hybridization buffer, DNA fragments below a certain size will fail to hybridize to the beads. As per the Broad institute protocol, 20% PEG, 2.5 M NaCl is added directly to the adapter ligation reaction at a final concentration of 0.3×, binding DNA fragments above 800 bp in size. The supernatant, which now contains DNA fragments below 800 bp in size is transferred to a new plate where 20% PEG 2.5 M NaCl is added at 1.2× volume to the supernatant, this time binding everything above approximately 100 bp to the SPRI beads. Supernatant is then discarded and beads are eluted with 30 μL of tris-HCl. Samples are now ready for PCR and addition of barcodes.

The SPRI methodology ultimately allows both column-free cleanup of samples and gel-free size selection, which makes it highly amenable to robust, large-scale multiplexing. In our experience, SPRI beads represent a costly but worthwhile initial investment for large scale GBS, but for smaller experiments a more standard column/gel protocol may be optimal. Finally, it is worth noting that sizing by SPRI concentration does not produce hard cutoffs. We initially attempted to fractionate ligation products with lower limit of 200 bp, corresponding to approximately 80 bp DNA fragment plus adaptors and an upper limit of 800 bp, or 680 bp of gDNA. Following sequencing, we observed significant DNA fragments below the expected size and a variable upper size limit for DNA fragments that tended to be below 680 bp. Little or no adaptor dimer contamination was observed.

### Barcoding and multiplexing

Following size fractionation, to amplify the sequenceable portion of the library as well as add barcodes for sample identification post multiplexing, we employed a six cycle PCR using KAPA HiFi Master Mix (KK2101, Kapa Biosystems) according to manufacturer’s instructions and primers described in Additional file 9: Table S2. PCR conditions were 95°C for 5 min followed by 6 cycles of 98°C for 20 sec, 65°C for 15 sec, and 72°C for 30 sec. Finally, 72°C for 1 min and 4°C hold. Following barcoding, SPRI beads were added at 1.5× concentration, and samples were washed per Broad Institute protocol then pooled.

Barcoding was performed using a dual-indexing system based on the TruSeq Dual Index Sequencing Primer Box that is further described in Lamble et al. [32]. While the TruSeq Dual-Index Sequencing Primer Box (FC-121-1003, Illumina) offers compatibility with up to 96 libraries, much higher levels of multiplexing are possible with custom primers. Lamble et al. offer a list of 120 indices that meet the necessary requirements. Base primer sequences, which incorporate the barcodes, are given in Additional file 9: Table S2 (Additional file 9: Table S2). Primers with custom indices should be selected with input from the user’s sequencing center to ensure compatibility with local protocols.

### DNA sequencing

The *O. sativa* and *Z. mays* digest sample libraries as well as the B73 × CG HincII and RsaI mapping population libraries were sequenced as paired-end 75 bp reads on the Illumina HiSeq 2500 according to manufacturer’s protocol. Image analysis and base calling was done using the Illumina version 1.8 pipeline with default parameters.

### Computational resources

Dataset analysis was performed on the Yale High Performance Computing Cluster. The YHPC clusters run a shared Linux environment with Perl ver 5.10.1, Python version 2.6.6, and Java version 1.7.0.

### Virtual restriction digest and associated data analysis

*In silico* restriction digests were performed on the *Z. mays* B73 (v2) [35] and *O. sativa* japonica Nipponbare 1.0 [36] reference genomes for all tested enzymes using a custom Python script that employed a sliding window algorithm. For MlyI, sites were identified on both the forward and reverse strands due to its non-palindromic recognition motif. Only reference positions that were a complete match to the recognition motif were recorded. The resulting digest map provided a framework for subsequent data analysis. Of interest for many downstream analyses were predicted sites, DNA fragments between proximal restriction sites. Predicted sites, due to their limited number compared to possible mispair (fragments generated from non-proximal restriction sites), singlet (fragments with only one end originating from a restriction site), and null (fragments with neither end originating from a restriction site), provided a useful control against sites with actual sequencing coverage for analyses of methylation, genic enrichment, GC bias, etc. Downstream analyses on the sequencing dataset and comparisons between aligned reads and predicted restriction sites were performed using custom Perl and Java scripts unless otherwise noted.

### Read alignment

Bowtie2 (parameters –N 1 –L 20 –D 20 –R 3 –I S,1,0.50) [60] was used to align *Z. mays* and *O. sativa* reads to the unmasked B73 reference genome and the Nipponbare *O. sativa* reference genome, respectively. These parameters were selected to maximize the probability of finding the correct alignment at the cost of increased runtime, which is especially important for the B73 genome given its high repetitive content.

### Genic enrichment and methylation sensitivity

Genic enrichment was determined by comparing the total set of predicted sites and predicted sites with sequencing coverage to gene databases for maize and rice. These datasets annotate give the positions of intronic, exonic, and untranslated sequence. For maize, the utilized dataset was the filtered, 5 b dataset (maizesequence.org) [35], which has transposases, pseudogenes, contamination, and low confidence events. The rice dataset was the IRGSP 1.0 reference dataset, which includes intronic, exonic, and untranslated sequence [36]. This dataset is supported by FL-cDNAs, ESTs, and proteins.

Methylation sensitivity was determined by comparing nucleotide frequencies around the set of total, predicted restriction sites to nucleotide frequencies around predicted sites with sequencing coverage. Differences between predicted and covered datasets in guanine ratios 1-2 bases upstream and cytosine ratios 1-2 bases downstream of restriction motifs were potentially due to methylation, as plant methylation can occur at CpG and CpNpG motifs. Changes in other nucleotide ratios were used to measure variance between predicted and covered sites not caused by methylation. The total set of predicted versus covered nucleotide ratios was further divided into genic and non-genic groups based on annotated datasets [35, 36].

### Variant calling

Variants were called from aligned reads using Samtools mpileup [61]. Variants retained in the final B73 × CG dataset were required to have Phred ≥30, MQ ≥30, homozygous, opposite states in the parentals, ≥2× coverage in 20 F_2_ samples, heterozygosity ≥0.2 and ≤0.8 in F2, and mean *r*^2^ correlation ≥0.3 five variants upstream or downstream (Additional file 10).

### Data imputation

Missing variant states were not directly imputed; instead, regions were classified as B73 homozygous, heterozygous, or CG homozygous. For this, variants were first phased by parental states, then a most likely state (B73 homozygous, CG homozygous, or heterozygous) was determined in 5 Mbp sliding window across the genome using a least squares based method. This method can be described using the equation:


Where *S* is the sum of residuals, and *r* is the residual defined by the equation:


Where *g*_*i*_ is the window genotype and _mi_ is the individual marker’s genotype. The three possible marker genotypes, homozygous B73, heterozygous, and homozygous CG were assigned values of 0, 1, and 2 respectively. Each possible “overall” genotype is assigned a value using the same system, and each of the three possible genotypes is tested against the set of markers. The genotype with the lowest sum of squared residuals is assigned to the window. In windows where less than ten total variants existed, variant states in proximal windows were included. Recombination breakpoints were resolved by first identifying proximal bins with differing calls. A five marker sliding window was then moved across the two proximal bins in a forward and reverse direction and a genotype call was obtained at each point. When the window transitioned from the first bin’s genotype to the second’s and vice versa, the point was recorded. Finally, the mean value of the two transition points was used as the point of recombination. This method was employed to resolve heterozygous regions in GBS data in spite of the high rate of missing and erroneous data, especially false homozygous calls resulting from low coverage of heterozygous SNPs.

### Trait mapping

Two traits (*y1* and *su1*) with previously mapped genetic positions segregated within the B73xCG F_2_ population. Genotypes of F_2_ individuals for both traits were determined based on the F_3_ endosperm phenotypes. Trait mapping was performed on pre and post-imputation datasets of filtered markers using a custom script utilizing the apache commons (commons.apache.org/math) implementation of the One-Way ANOVA test.

### Resampling of RsaI and HincII datasets

One RsaI sample (F_2_-44) and one HincII sample (F_2_-23) were selected from the B73xCG F_2_ population and subsets of reads were randomly subsampled from each dataset. For RsaI, reads were subsampled in 100,000 read intervals from 100,000 reads to 2,000,000 reads, in 200,000 read intervals from 2,000,000 reads to 3,000,000 reads, and in 500,000 read intervals from 3,000,000 reads to 7,000,000 reads. The original sample had 15,389,878 2 × 75 bp reads. For HincII, reads were subsampled at 30,000, 40,000, and 50,000 reads and from 100,000 to 3,000,000 reads in 100,000 read intervals. The original sample (F_2_-23), had 3,968,544 2 × 75 bp reads. Subsamplings were done to cover the range of diminishing returns for additional markers. The lowest value for each sample was determined by the point at which imputation would fail due to too few markers. Each subset was independently aligned to the genome, variant calling and filtering applied, and finally genotypes were imputed. To evaluate the subsamples, the number of shared, post-filter markers was compared between the original sample and the subsets. In addition, the fraction of the genome that shared the same call between the subset and the original was determined.

### Availability of supporting data

The datasets supporting the results of this article are included are included within the article as Additional file 10.

### Ethics

No research involving human subjects, human data, or regulated vertebrates or invertebrates was included in this study.

## Electronic supplementary material

Additional file 1: **Coverage distributions by predicted site size for all tested enzymes.** Predicted sites with sequencing coverage were binned first by size and then by depth of coverage for all enzymes tested with A) maize and B) rice. All sites with depths of coverage >100× were binned at 100×. (PDF 836 KB)

Additional file 2: **GC content of covered versus predicted sites between 100-200bp.** To test the effect of GC content on sequencing coverage, the GC content of total predicted sites between 100 and 200 bp was compared to the GC content of predicted sites with sequencing coverage normalized by depth of coverage for A) maize and B) rice. (PDF 113 KB)

Additional file 3: **Fraction of predicted sites with aligned reads versus total predicted sites in genic regions.** The A) maize and B) rice genomes were binned into 1 Mbp intervals, then within each bin the fraction of covered sites in genic regions was compared to the fraction of predicted sites in genic regions. Bins were then plotted based on the two ratios and the number of bins in a given point indicated via heatmap. The white lines are present to indicate the predicted values at which the covered and predicted genic fractions would be identical. Points above this line represent bins with a greater fraction of sequenced sites in genic regions than predicted. (PDF 279 KB)

Additional file 4: **Inferred methylation sensitivity of restriction enzymes.** Methylation sensitivity was inferred through changes between predicted and covered sites one to two bases upstream and guanine one to two bases downstream for A) maize and B) rice. Error bars represent two standard deviations based on nucleotide ratios three through twelve bases upstream and downstream. (PDF 358 KB)

Additional file 5: **Summary information for F**_**2**_ **B73 × CG cross population.** (XLSX 15 KB)

Additional file 6: **Raw GBS HincII dataset from an F**_**2**_ **admixture population.** Post-filter, parental-phased variants from the B73 × CG HincII F_2_ dataset were paced in 5 Mbp bins spanning the maize genome. Bin heatmaps indicate “mean genotype” value of variants within in the bin. Sample order is given, outermost to innermost, in Additional file 5: Table S1. (PDF 4 MB)

Additional file 7: **Imputed GBS HincII dataset from an F**_**2**_ **admixture population.** Sample order is given, outermost to innermost, in Additional file 5: Table S1. (PDF 3 MB)

Additional file 8: **Comparison RsaI and HincII F**_**2**_ **Imputed GBS datasets.** Randomly selected samples processed by both RsaI and HincII in independent experiments are displayed as paired rings. The RsaI dataset is the outer ring, and the HincII dataset is the inner ring of each pair. From outermost to innermost, the displayed samples are F_2_-82, F_2_-35, F_2_-51, F_2_-44, F_2_-62, F_2_-39, F_2_-30, F_2_-63. (PDF 1 MB)

Additional file 9: **Library preparation oligo sequences.** (XLSX 43 KB)

Additional file 10: **Filtered VCF files for B73xCG F**_**2**_ **populations.** Modified mpileup VCF format files provide the filtered RsaI and HincII B73xCG F2 datasets. Removed sample calls are given as “X”. Retained sample calls are displayed as colon separated genotype and depth of coverage. Standard information on variant position, reference and alternate allele, and quality metrics are retained. Non-independent markers are retained. (ZIP 8 MB)

## Abbreviations

WGS: Whole genome sequencing
RRS: Reduced representation sequencing
RAD: Restriction site Associated DNA markers
GBS: Genotyping-by-Sequencing
CG: Country gentleman (maize inbred)
EST: Expressed sequence tag
PEG: Polyethylene Glycol.
