# Supplementary material for: Flexible and scalable genotyping-by-sequencing strategies for population studies
Source: BMC Genomics. 2014 Nov 18;15(1):979. doi: 10.1186/1471-2164-15-979 (PMC4253001; doi:10.1186/1471-2164-15-979)
Supplement: Supplementary file 1 — Additional file 1: Coverage distributions by predicted site size for all tested enzymes. Predicted sites with sequencing coverage were binned first by size and then by depth of coverage for all enzymes tested with A) maize and B) rice. All sites with depths of coverage >100× were binned at 100×. (PDF 836 KB) [file 12864_2014_6697_MOESM1_ESM.pdf]

a

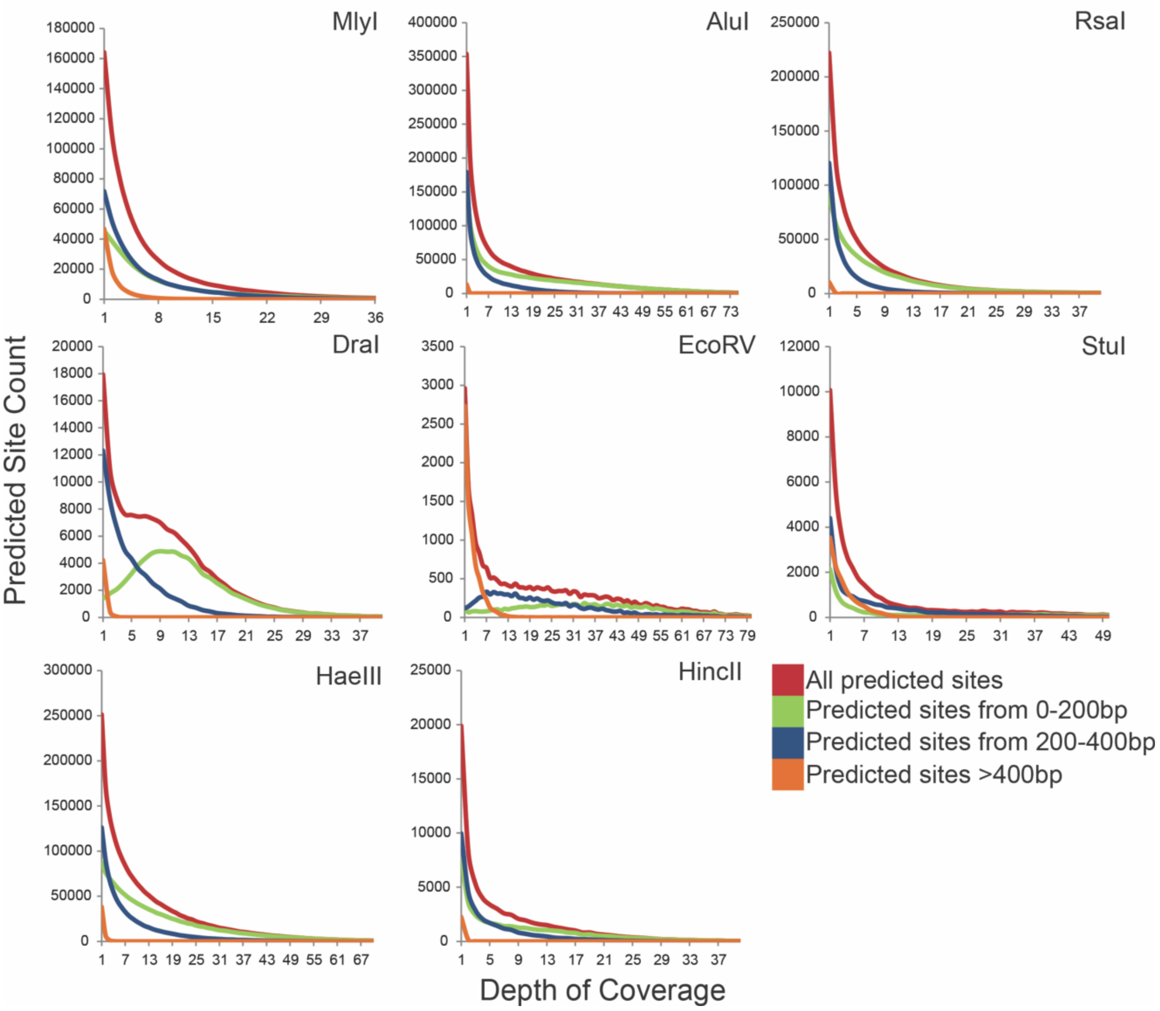

MlyI
AluI
RsaI
DraI
EcoRV
StuI
HaeIII
HincII
Predicted Site Count
Depth of Coverage
All predicted sites
Predicted sites from 0-200bp
Predicted sites from 200-400bp
Predicted sites >400bp

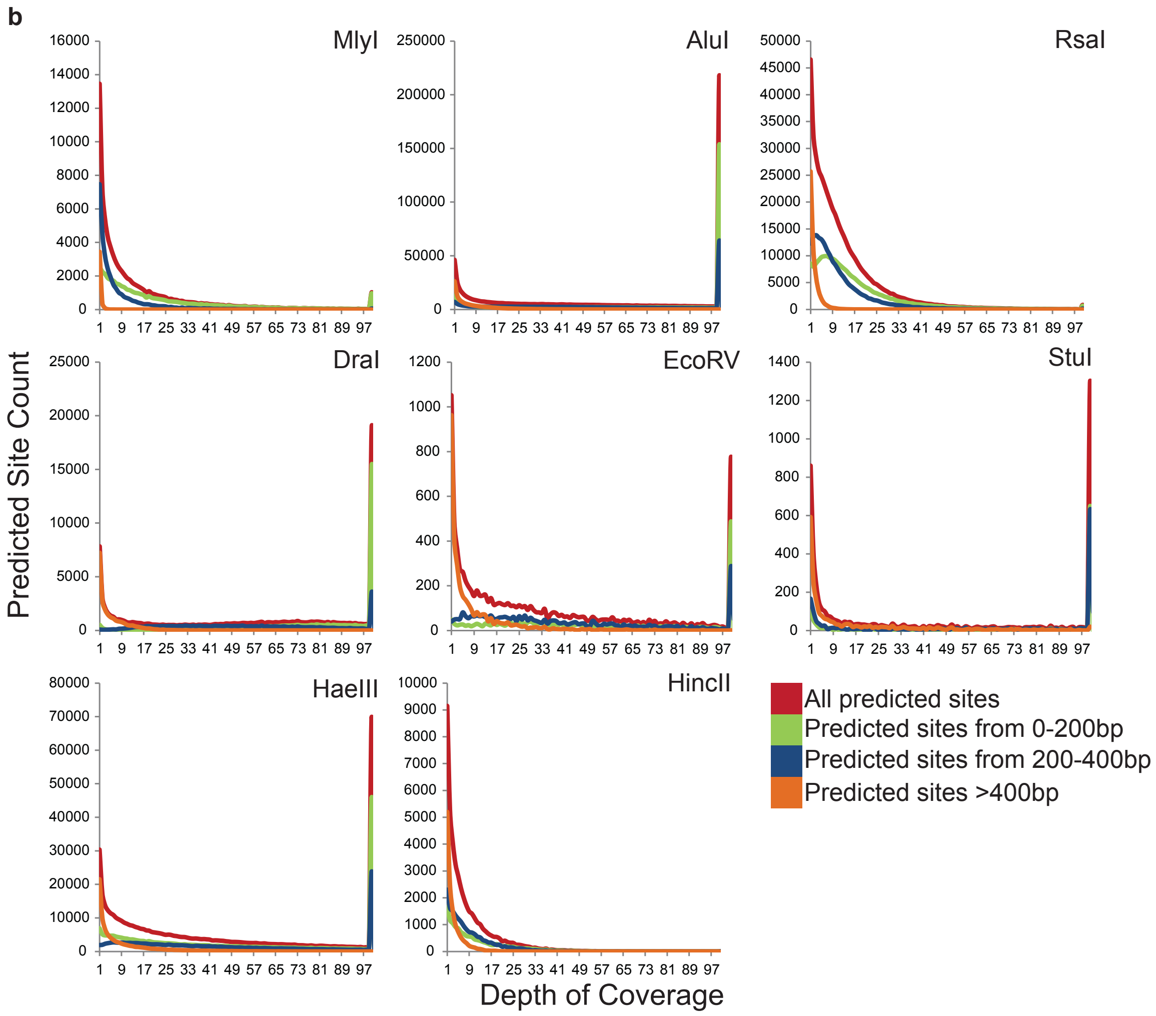

Additional File 1 Supplementary Figure 1: Coverage distributions by predicted site size for all tested enzymes.

Predicted sites with sequencing coverage were binned first by size and then by depth of coverage for all enzymes tested with A) maize and B) rice. All sites with depths of coverage > 100x were binned at 100x.
