# Supplementary material for: Flexible and scalable genotyping-by-sequencing strategies for population studies
Source: BMC Genomics. 2014 Nov 18;15(1):979. doi: 10.1186/1471-2164-15-979 (PMC4253001; doi:10.1186/1471-2164-15-979)
Supplement: Supplementary file 2 — Additional file 2: GC content of covered versus predicted sites between 100-200bp. To test the effect of GC content on sequencing coverage, the GC content of total predicted sites between 100 and 200 bp was compared to the GC content of predicted sites with sequencing coverage normalized by depth of coverage for A) maize and B) rice. (PDF 113 KB) [file 12864_2014_6697_MOESM2_ESM.pdf]

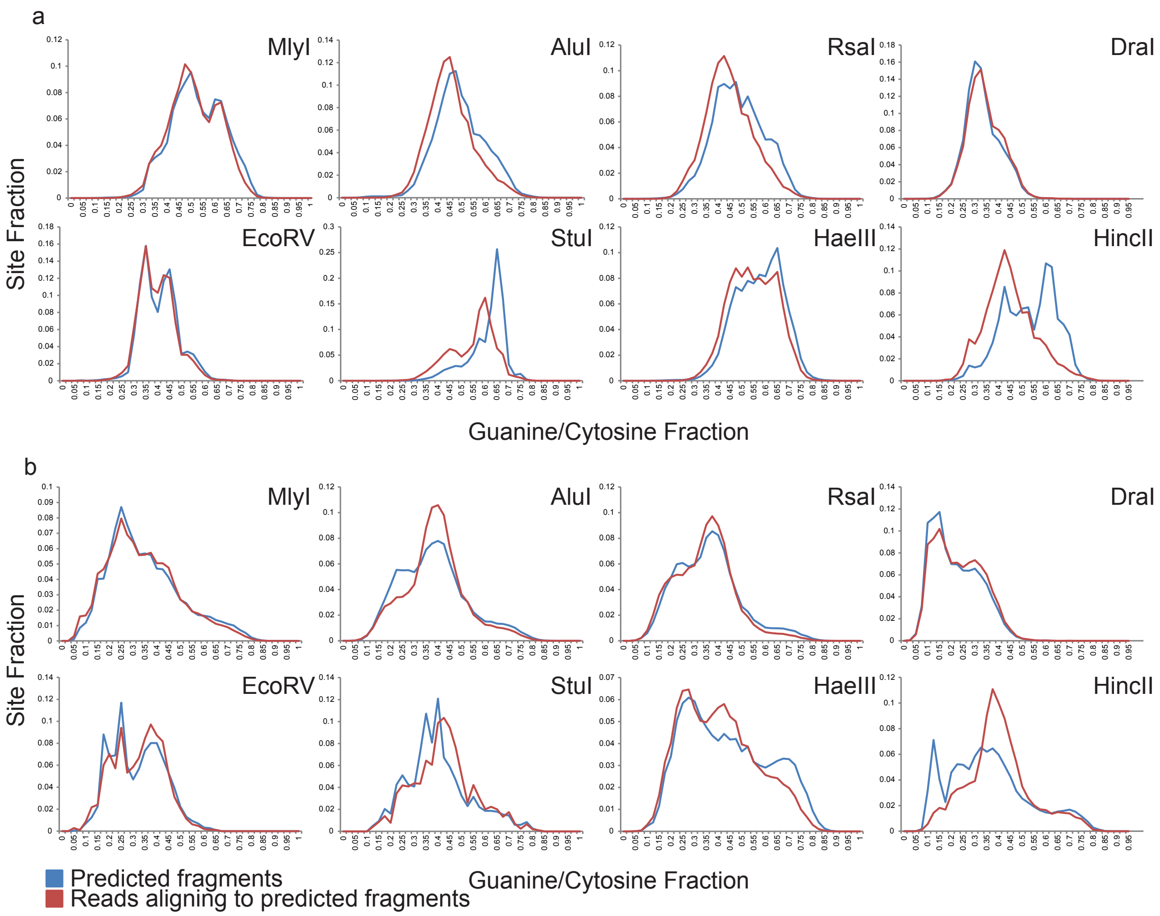

Additional File 2 Supplementary Figure 2: GC content of covered versus predicted sites between 100-200 bp.

To test the effect of GC content on sequencing coverage, the GC content of total predicted sites between 100 and 200 bp was compared to the GC content of predicted sites with sequencing coverage normalized by depth of coverage for A) maize and B) rice.
