# Supplementary material for: Flexible and scalable genotyping-by-sequencing strategies for population studies
Source: BMC Genomics. 2014 Nov 18;15(1):979. doi: 10.1186/1471-2164-15-979 (PMC4253001; doi:10.1186/1471-2164-15-979)
Supplement: Supplementary file 3 — Additional file 3: Fraction of predicted sites with aligned reads versus total predicted sites in genic regions. The A) maize and B) rice genomes were binned into 1 Mbp intervals, then within each bin the fraction of covered sites in genic regions was compared to the fraction of predicted sites in genic regions. Bins were then plotted based on the two ratios and the number of bins in a given point indicated via heatmap. The white lines are present to indicate the predicted values at which the covered and predicted genic fractions would be identical. Points above this line represent bins with a greater fraction of sequenced sites in genic regions than predicted. (PDF 279 KB) [file 12864_2014_6697_MOESM3_ESM.pdf]

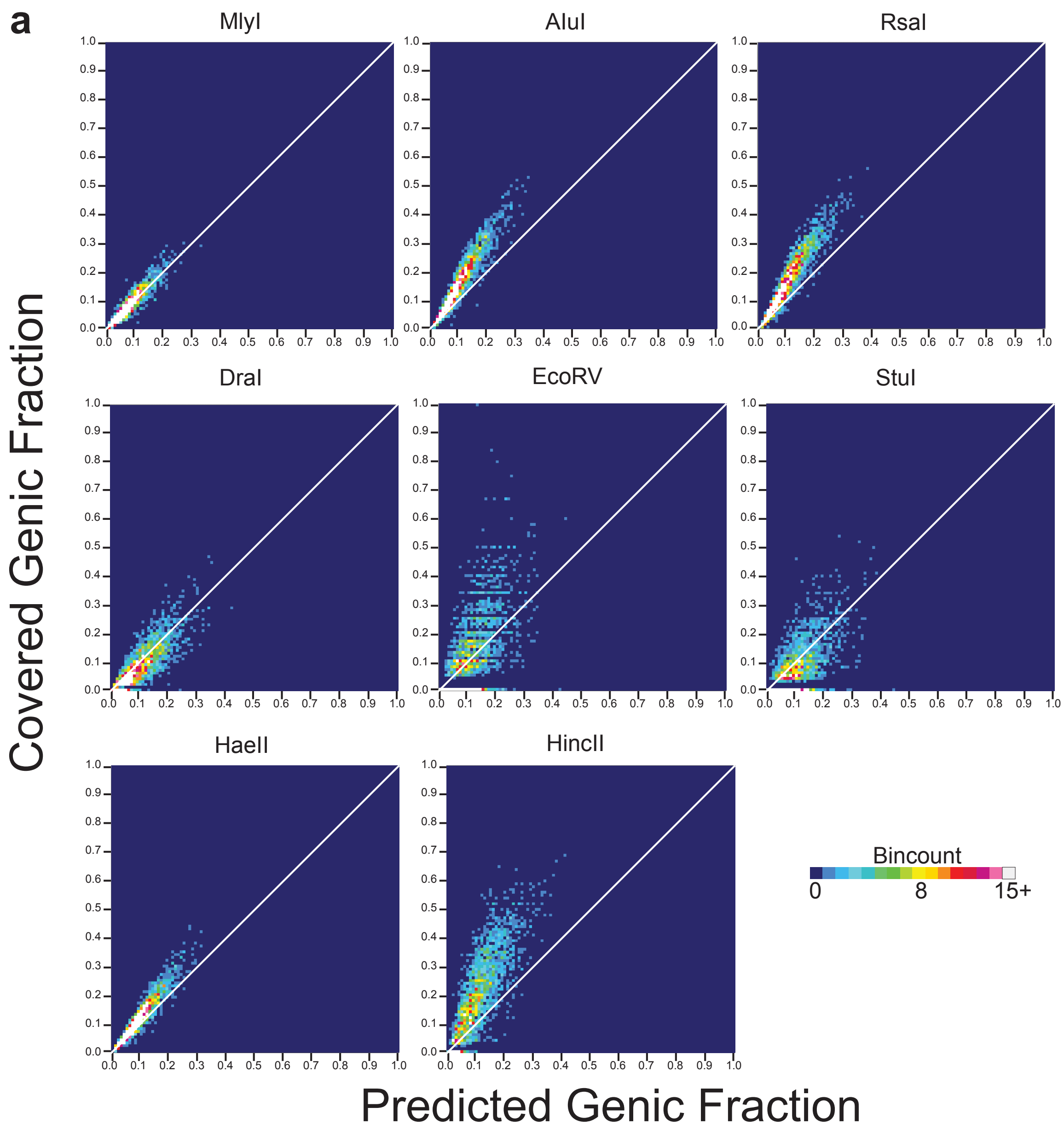

a
MlyI
AluI
RsaI
DraI
EcoRV
StuI
HaeII
HincII
Bincount
0
8
15+
Covered Genic Fraction
Predicted Genic Fraction

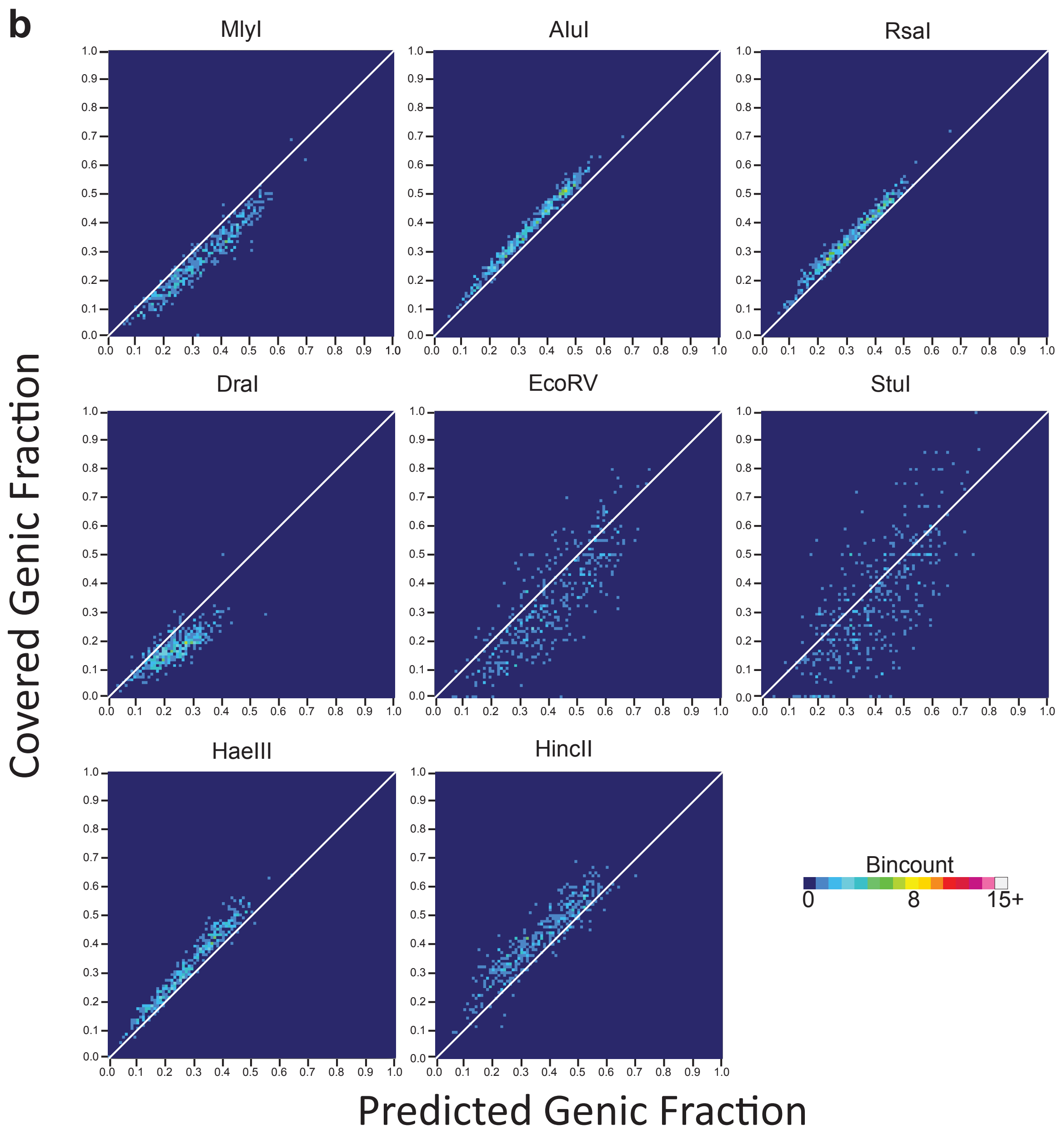

Additional File 3 Supplementary Figure 3: Fraction of predicted sites with aligned reads versus total predicted sites in genic regions.

The A) maize and B) rice genomes were binned into 1 Mbp intervals, then within each bin the fraction of covered sites in genic regions was compared to the fraction of predicted sites in genic regions. Bins were then plotted based on the two ratios and the number of bins in a given point indicated via heatmap. The white lines are present to indicate the predicted values at which the covered and predicted genic fractions would be identical. Points above this line represent bins with a greater fraction of sequenced sites in genic regions than predicted.
