# Supplementary material for: Flexible and scalable genotyping-by-sequencing strategies for population studies
Source: BMC Genomics. 2014 Nov 18;15(1):979. doi: 10.1186/1471-2164-15-979 (PMC4253001; doi:10.1186/1471-2164-15-979)
Supplement: Supplementary file 4 — Additional file 4: Inferred methylation sensitivity of restriction enzymes. Methylation sensitivity was inferred through changes between predicted and covered sites one to two bases upstream and guanine one to two bases downstream for A) maize and B) rice. Error bars represent two standard deviations based on nucleotide ratios three through twelve bases upstream and downstream. (PDF 358 KB) [file 12864_2014_6697_MOESM4_ESM.pdf]

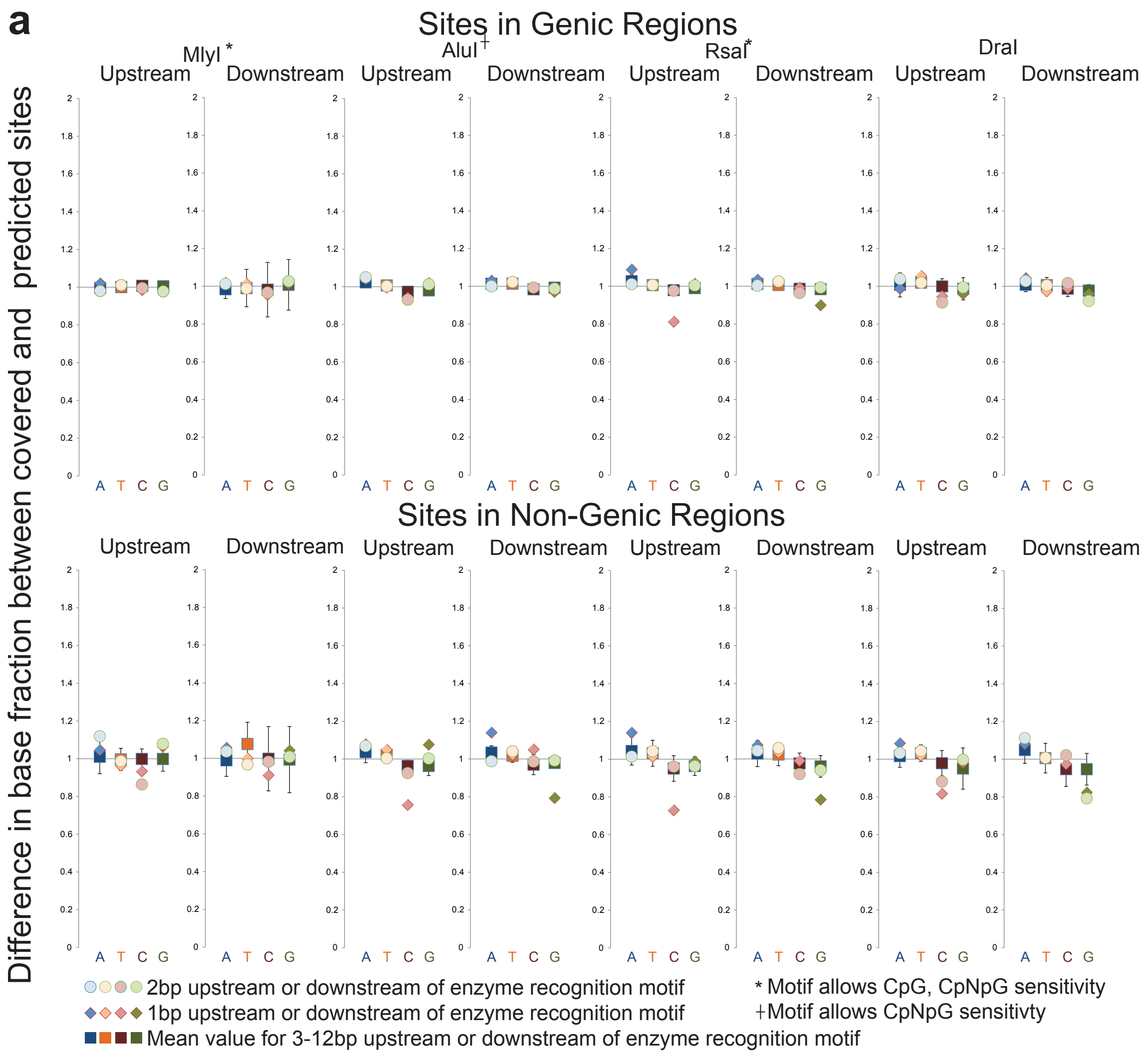
a
Sites in Genic Regions
MlyI *
AluI +
RsaI *
DraI
Upstream
Downstream
Sites in Non-Genic Regions
A T C G
Difference in base fraction between covered and predicted sites
2bp upstream or downstream of enzyme recognition motif
1bp upstream or downstream of enzyme recognition motif
Mean value for 3-12bp upstream or downstream of enzyme recognition motif
* Motif allows CpG, CpNpG sensitivity
+Motif allows CpNpG sensitivty

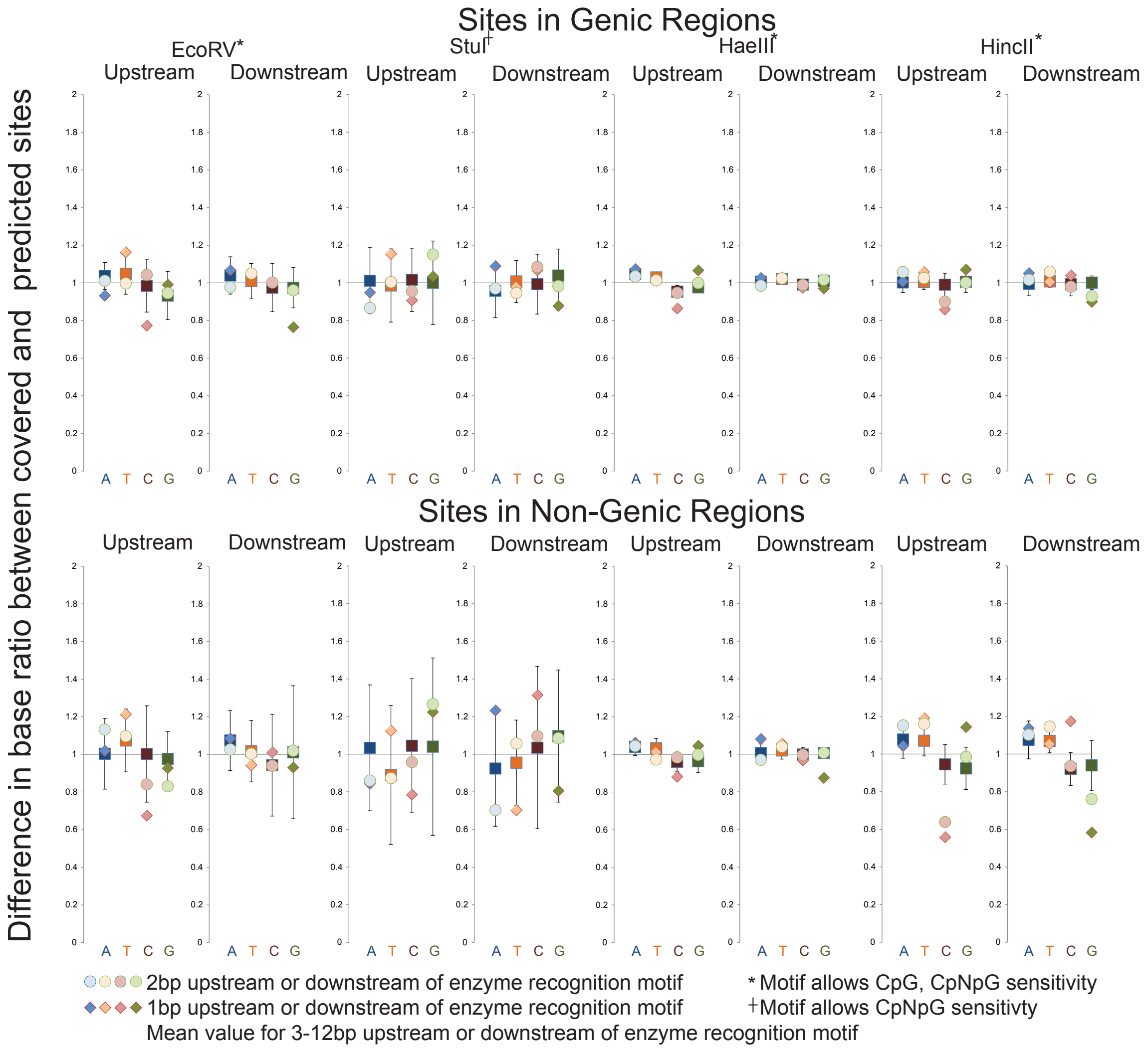

Sites in Genic Regions
EcoRV*
StuI┼
HaeIII*
HincII*
Upstream
Downstream
Sites in Non-Genic Regions
Difference in base ratio between covered and predicted sites
A T C G
2bp upstream or downstream of enzyme recognition motif
1bp upstream or downstream of enzyme recognition motif
Mean value for 3-12bp upstream or downstream of enzyme recognition motif
* Motif allows CpG, CpNpG sensitivity
┼Motif allows CpNpG sensitivty

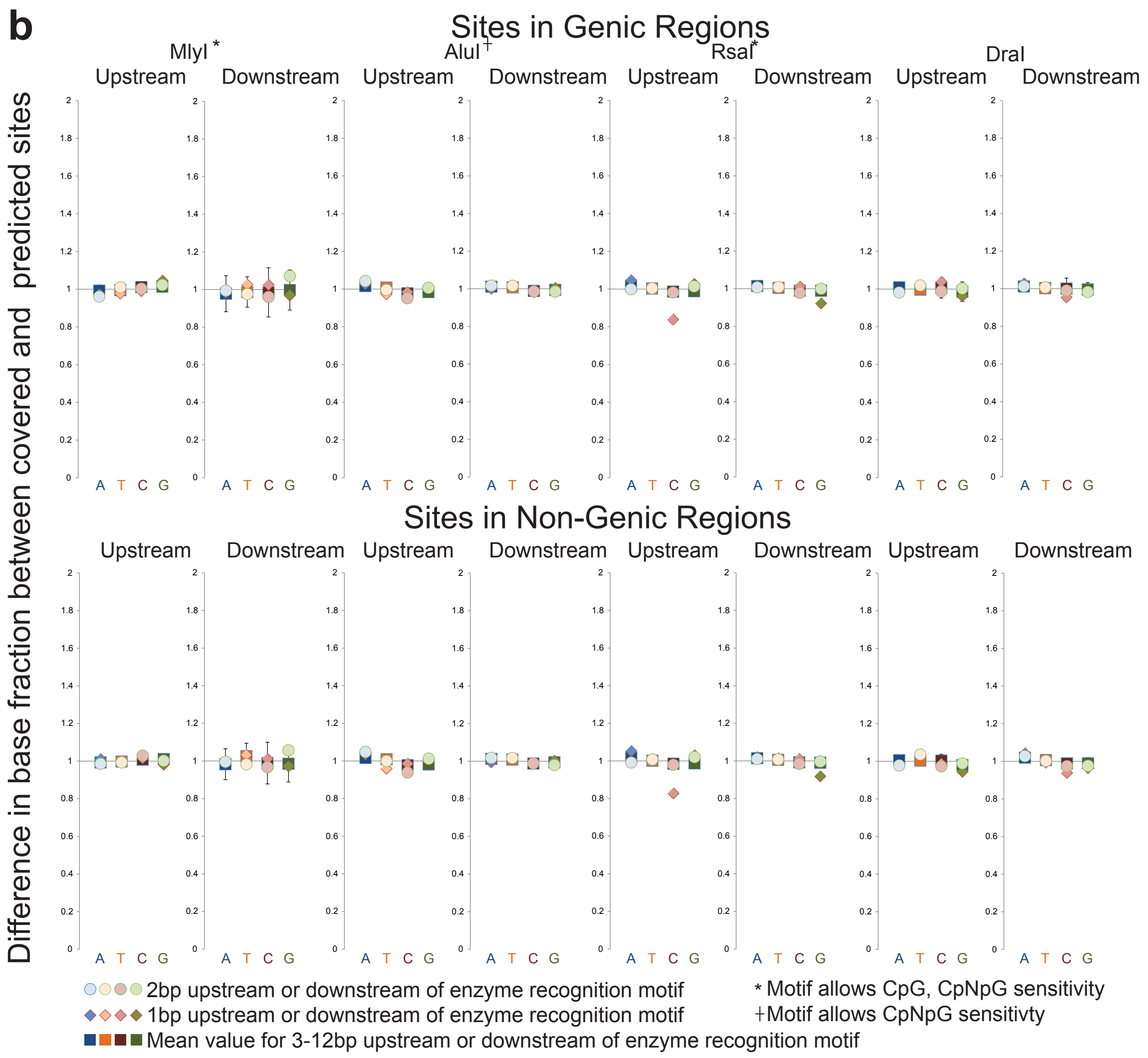
b
Sites in Genic Regions
MlyI *
AluI †
RsaI *
DraI
Upstream
Downstream
Sites in Non-Genic Regions
A T C G
Difference in base fraction between covered and predicted sites
2bp upstream or downstream of enzyme recognition motif
1bp upstream or downstream of enzyme recognition motif
Mean value for 3-12bp upstream or downstream of enzyme recognition motif
* Motif allows CpG, CpNpG sensitivity
†Motif allows CpNpG sensitivty

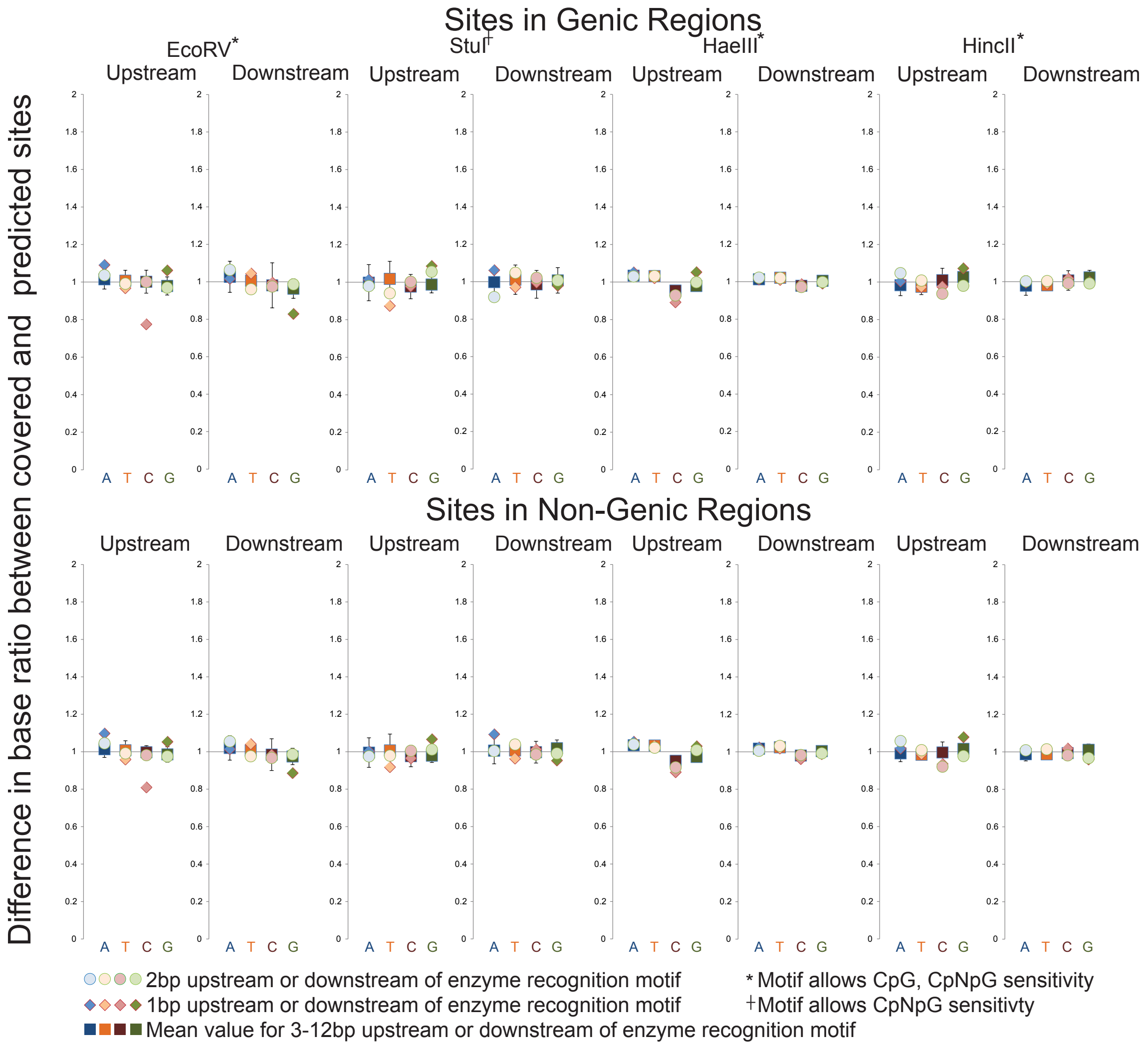

Additional File 4 Supplementary Figure 4: Inferred methylation sensitivity of restriction enzymes.

Methylation sensitivity was inferred through changes between predicted and covered sites one to two bases upstream and guanine one to two bases downstream for A) maize and B) rice. Error bars represent two standard deviations based on nucleotide ratios three through twelve bases upstream and downstream.
