# Supplementary material for: Flexible and scalable genotyping-by-sequencing strategies for population studies
Source: BMC Genomics. 2014 Nov 18;15(1):979. doi: 10.1186/1471-2164-15-979 (PMC4253001; doi:10.1186/1471-2164-15-979)
Supplement: Supplementary file 6 — Additional file 6: Raw GBS HincII dataset from an F 2 admixture population. Post-filter, parental-phased variants from the B73 × CG HincII F2 dataset were paced in 5 Mbp bins spanning the maize genome. Bin heatmaps indicate “mean genotype” value of variants within in the bin. Sample order is given, outermost to innermost, in Additional file 5: Table S1. (PDF 4 MB) [file 12864_2014_6697_MOESM6_ESM.pdf]

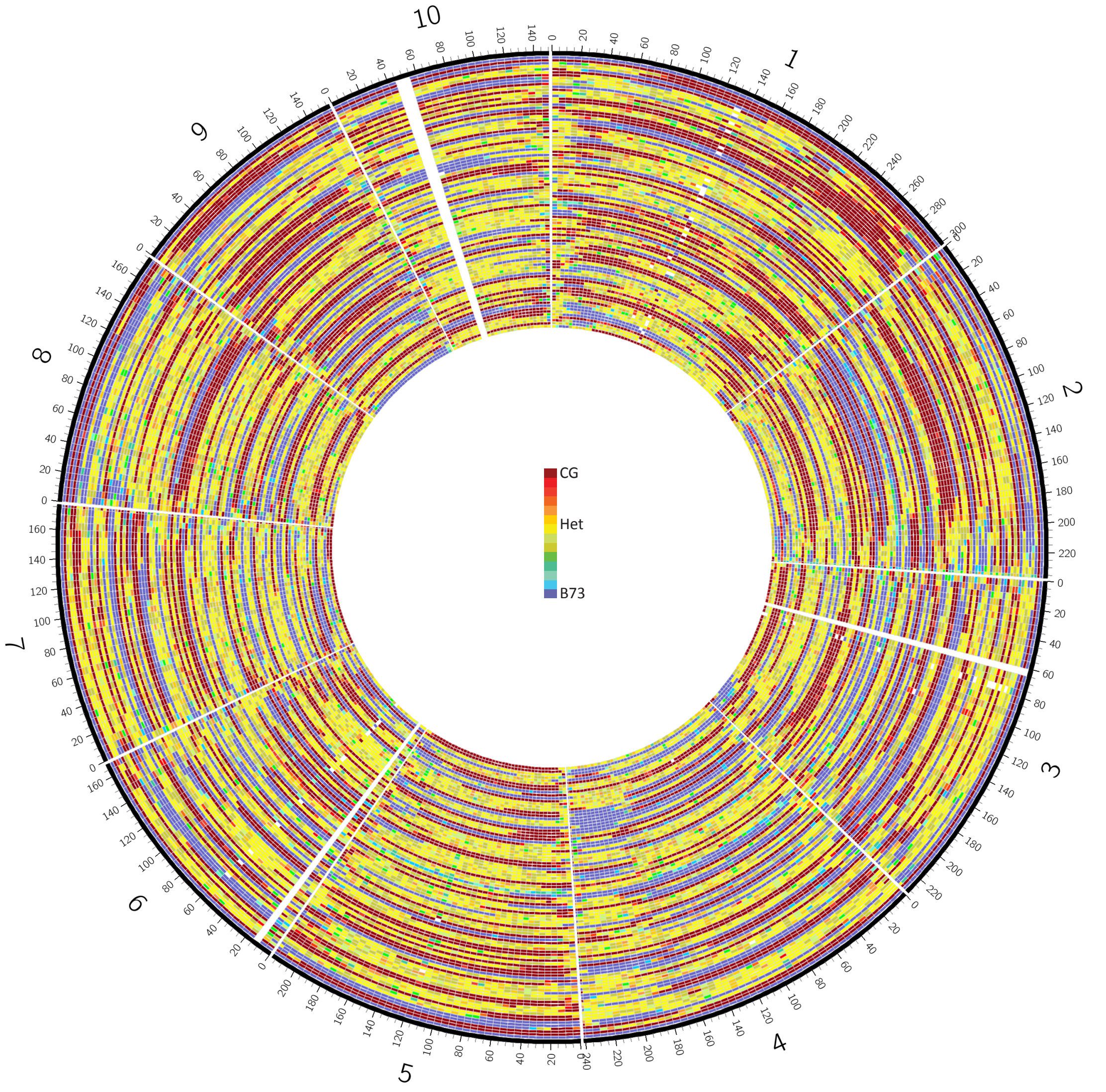

Additional File 6 Supplementary Figure 5: Raw GBS HincII dataset from an F_2 admixture population.

Post-filter, parental-phased variants from the B73 x CG HincII F_2 dataset were paced in 5 Mbp bins spanning the maize genome. Bin heatmaps indicate "mean genotype" value of variants within in the bin. Sample order is given, outermost to innermost, in Additional File 5, Supplementary Table 1.
