# Supplementary material for: Flexible and scalable genotyping-by-sequencing strategies for population studies
Source: BMC Genomics. 2014 Nov 18;15(1):979. doi: 10.1186/1471-2164-15-979 (PMC4253001; doi:10.1186/1471-2164-15-979)
Supplement: Supplementary file 7 — Additional file 7: Imputed GBS HincII dataset from an F 2 admixture population. Sample order is given, outermost to innermost, in Additional file 5: Table S1. (PDF 3 MB) [file 12864_2014_6697_MOESM7_ESM.pdf]

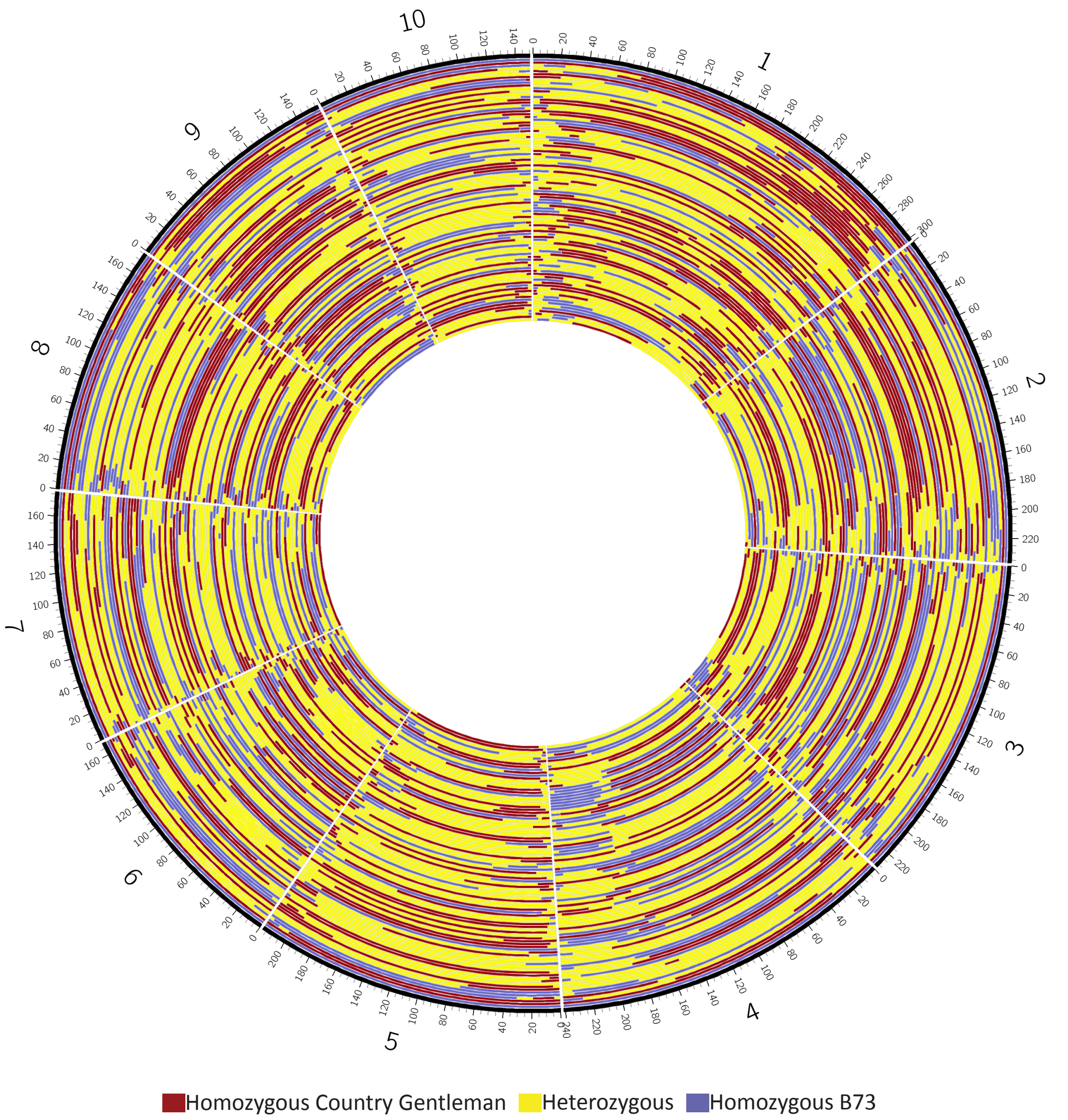

Additional File 7 Supplementary Figure 6: Imputed GBS HincII dataset from an F_2 admixture population.

Sample order is given, outermost to innermost, in Additional File 6, Supplementary Table 1.
