# Supplementary material for: Flexible and scalable genotyping-by-sequencing strategies for population studies
Source: BMC Genomics. 2014 Nov 18;15(1):979. doi: 10.1186/1471-2164-15-979 (PMC4253001; doi:10.1186/1471-2164-15-979)
Supplement: Supplementary file 8 — Additional file 8: Comparison RsaI and HincII F 2 Imputed GBS datasets. Randomly selected samples processed by both RsaI and HincII in independent experiments are displayed as paired rings. The RsaI dataset is the outer ring, and the HincII dataset is the inner ring of each pair. From outermost to innermost, the displayed samples are F2-82, F2-35, F2-51, F2-44, F2-62, F2-39, F2-30, F2-63. (PDF 1 MB) [file 12864_2014_6697_MOESM8_ESM.pdf]

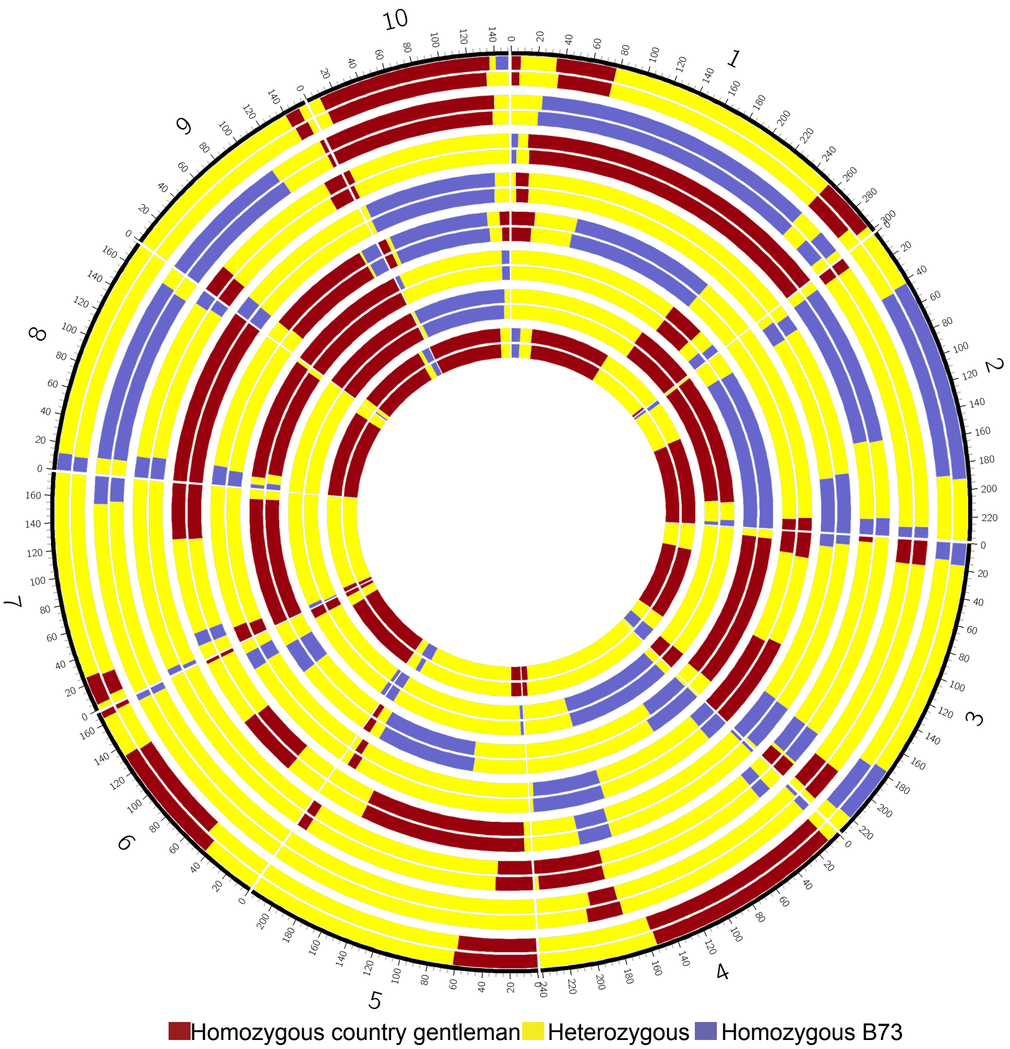

Additional File 8 Supplementary Figure 7: Comparison RsaI and HincII F_2 Imputed GBS datasets.

Randomly selected samples processed by both RsaI and HincII in independent experiments are displayed as paired rings. The RsaI dataset is the outer ring, and the HincII dataset is the inner ring of each pair. From outermost to innermost, the displayed samples are F_2-82, F_2-35, F_2-51, F_2-44, F_2-62, F_2-39, F_2-30, F_2-63.
